# Large-Scale Production of Human iPSC-Derived Macrophages for Drug Screening

**DOI:** 10.3390/ijms21134808

**Published:** 2020-07-07

**Authors:** Simon Gutbier, Florian Wanke, Nadine Dahm, Anna Rümmelin, Silke Zimmermann, Klaus Christensen, Fabian Köchl, Anna Rautanen, Klas Hatje, Barbara Geering, Jitao David Zhang, Markus Britschgi, Sally A. Cowley, Christoph Patsch

**Affiliations:** 1 Roche Pharma Research and Early Development, Therapeutic Modalities, Roche Innovation Center Basel, F. Hoffmann-La Roche Ltd., Grenzacherstrasse 124, 4070 Basel, Switzerland; Nadine.Dahm@roche.com (N.D.); Anna.Ruemmelin@roche.com (A.R.); Silke.Zimmermann@roche.com (S.Z.); Klaus.Christensen@roche.com (K.C.); patschc@gmail.com (C.P.); 2 Roche Pharma Research and Early Development, Pharmaceutical Sciences, Roche Innovation Center Basel, F. Hoffmann-La Roche Ltd., Grenzacherstrasse 124, 4070 Basel, Switzerland; Fabian.Koechel@roche.com (F.K.); Anna.Rautanen@roche.com (A.R.); Klas.Hatje@roche.com (K.H.); jitao_david.zhang@roche.com (J.D.Z.); 3 Roche Pharma Research and Early Development, Neuroscience and Rare Diseases Discovery and Translational Area, Roche Innovation Center Basel, F. Hoffmann-La Roche Ltd., Grenzacherstrasse 124, 4070 Basel, Switzerland; markus.britschgi@roche.com; 4 Roche Pharma Research and Early Development, Immunology and Infectious Diseases Discovery and Translational Area, Roche Innovation Center Basel, F. Hoffmann-La Roche Ltd., Grenzacherstrasse 124, 4070 Basel, Switzerland; Florian.Wanke@roche.com (F.W.); Barbara.Geering@roche.com (B.G.); 5 James Martin Stem Cell Facility, Sir William Dunn School of Pathology, University of Oxford, Oxford OX1 3RE, UK; sally.cowley@path.ox.ac.uk; 6 BlueRock Therapeutics, New York, NY 10016, USA

**Keywords:** myeloid cells, primitive macrophages, induced pluripotent stem cells, in vitro characterization, drug discovery, disease modeling

## Abstract

Tissue-resident macrophages are key players in inflammatory processes, and their activation and functionality are crucial in health and disease. Numerous diseases are associated with alterations in homeostasis or dysregulation of the innate immune system, including allergic reactions, autoimmune diseases, and cancer. Macrophages are a prime target for drug discovery due to their major regulatory role in health and disease. Currently, the main sources of macrophages used for therapeutic compound screening are primary cells isolated from blood or tissue or immortalized or neoplastic cell lines (e.g., THP-1). Here, we describe an improved method to employ induced pluripotent stem cells (iPSCs) for the high-yield, large-scale production of cells resembling tissue-resident macrophages. For this, iPSC-derived macrophage-like cells are thoroughly characterized to confirm their cell identity and thus their suitability for drug screening purposes. These iPSC-derived macrophages show strong cellular identity with primary macrophages and recapitulate key functional characteristics, including cytokine release, phagocytosis, and chemotaxis. Furthermore, we demonstrate that genetic modifications can be readily introduced at the macrophage-like progenitor stage in order to interrogate drug target-relevant pathways. In summary, this novel method overcomes previous shortcomings with primary and leukemic cells and facilitates large-scale production of genetically modified iPSC-derived macrophages for drug screening applications.

## 1. Introduction

Macrophages are key players in inflammatory processes, and their activation and functionality are crucial in health and disease [1,2,3,4]. Diseases with confirmed macrophage involvement encompass metabolic diseases; allergic disorders; autoimmunity; chronic inflammatory diseases; cancer; neurodegenerative diseases; as well as bacterial, viral, parasitic, and fungal infections. Macrophages are widely distributed throughout tissues. While very often building the first line of immune defense in a disease context, macrophages are essential in the repair and homeostasis of the affected tissue as well. Therefore, impaired macrophage functionality and the loss of homeostasis are closely linked to the pathogenesis of degenerative diseases [5].

Key macrophage functions include phagocytosis (e.g., of pathogens, cellular debris, and dead cells), migration (to the site of damage), antigen presentation, as well as cytokine and soluble factor release to trigger further inflammatory responses or to render trophic support to the surrounding tissue [1,2,3,4]. For this reason, the pharmacologic modulation of monocyte/macrophage function reflects a therapeutic strategy to resolve many disease processes. The broad range of disease areas with macrophage involvement and the versatile functional properties of macrophages result in a vast number of potential drug targets [6]. Consequently, this generates a high demand for large numbers of macrophages in order to perform disease modeling and compound screening.

To date, macrophage research and employment in drug screening has been compromised by the limited availability of large uniform batches of authentic cells. One way to obtain macrophages is to isolate monocytes from PBMCs (peripheral blood mononuclear cells) enriched from blood draws, followed by in vitro differentiation to macrophages. However, limited cell numbers per donor, donor-to-donor variation, and limited genetic engineering possibilities restrict the use of these primary cells. Furthermore, these primary PBMC-derived cells resemble a non-tissue-resident, inflammatory and invasive blood-derived macrophage phenotype due to their ontogeny [7]. Therefore, in order to obtain tissue-resident macrophages, for example, microglia, the only option has been isolation from primary tissue, very often from mice. The murine origin of such macrophages comes with the additional downside of the need for high animal numbers and limited compatibility between the murine and human immune systems [8,9,10]. Other sources of monocyte-like cells include the leukemic THP-1 cells, which can be easily cultured and stored in liquid nitrogen in large batches. Although of the same lineage, the shortcomings are its cancer origin, which renders the cells more susceptible to spontaneous mutations, and its lack or lower expression of certain surface or cytoplasmic immunoglobulins compared to primary monocytes/macrophages [11,12,13].

Recent studies successfully derived macrophage-like progenitor cells from human-induced Pluripotent Stem Cells (iPSCs). These cells can be further specialized, either by co-culturing with other tissue specific cell types or by simulating tissue environment with soluble factors, to cells that resemble key characteristics of tissue-resident macrophages (for simplicity, herein called “iPSC-derived macrophages”) [14,15,16,17,18,19]. In mouse embryonic development Myb-independent macrophages from the primitive streak populate most tissues before the start of definitive hematopoiesis [20,21,22,23,24,25] and renew in most tissues with only minor contributions from adult blood derived monocytes [26,27]. Interestingly, most of the iPSC-based differentiation protocols resemble Myb-independent primitive myelopoiesis, also shown for the one improved in this study by Buchrieser et al. [28]. Hence, they are considered to be well suited for the generation of iPSC macrophages that model tissue resident macrophages of various tissues [7,29], displaying tissue-specific markers and functionality when compared to their primary counterparts [10,17,18,19,28,30]. Moreover, the method gives access to cells with a disease-relevant genetic background and readily allows genetic engineering (e.g., correction or introduction of a disease-causing mutation in the pluripotent state). The iPSC technology offers a virtually unlimited supply of in vitro-generated macrophages with consistent genotype and function, eliminating the risk of donor variability where needed. However, scaling up the numbers of iPSC-derived macrophages with uniform batches and stable phenotype towards high-throughput compound screening for drug discovery remains an issue.

Here, we describe an improved method for the high-yield, large-scale production, and intermediate storage of human iPSC-macrophage precursors and provide evidence of the applicability of these cells for drug screening and drug developmental purposes.

## 2. Results

### 2.1. Differentiation and Storage of Macrophage Progenitors from iPSCs

**Figure 1:**
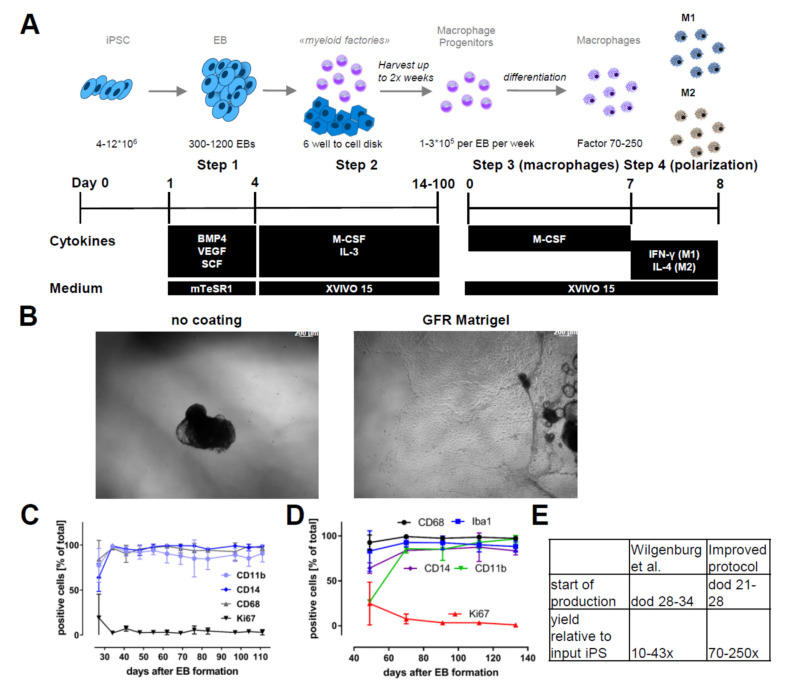
Differentiation protocol and robustness: (**A**) A differentiation scheme indicating the different steps, including cytokines, growth factors, and medium conditions to obtain macrophage progenitors and macrophages from induced pluripotent stem cells (iPSCs). (**B**) Representative phase images of embryoid body (EB) adherence to uncoated and growth factor-reduced (GFR) Matrigel-coated dishes. (**C**) Myeloid marker genes and proliferation marker of macrophage progenitors sampled over the complete production period of a myeloid factory (*n* = 3; iPSC lines SFC840-03-01 (STBCi026-B), SFC831-03-03 (STBCi024-B), and SBNEO1). (**D**) Marker gene expression (CD68, IBA1, CD14, and CD11b) in macrophages differentiated from progenitors harvested at different time points of blood factory lifecycle (*n* = 3; iPSC line SFC840-03-01). (**E**) Comparison of differentiation times until start of macrophage precursor production and yields per input iPSC from the original protocol [31] and the modified version presented here. Differentiation protocols were tested in this study with iPSC lines SFC840-03-01 (STBCi026-B), SFC831-03-03 (STBCi024-B), SBNeo1, and SBAD3-01 and with Bioneer C10 (H266 C10 GC).

**Figure 2:**
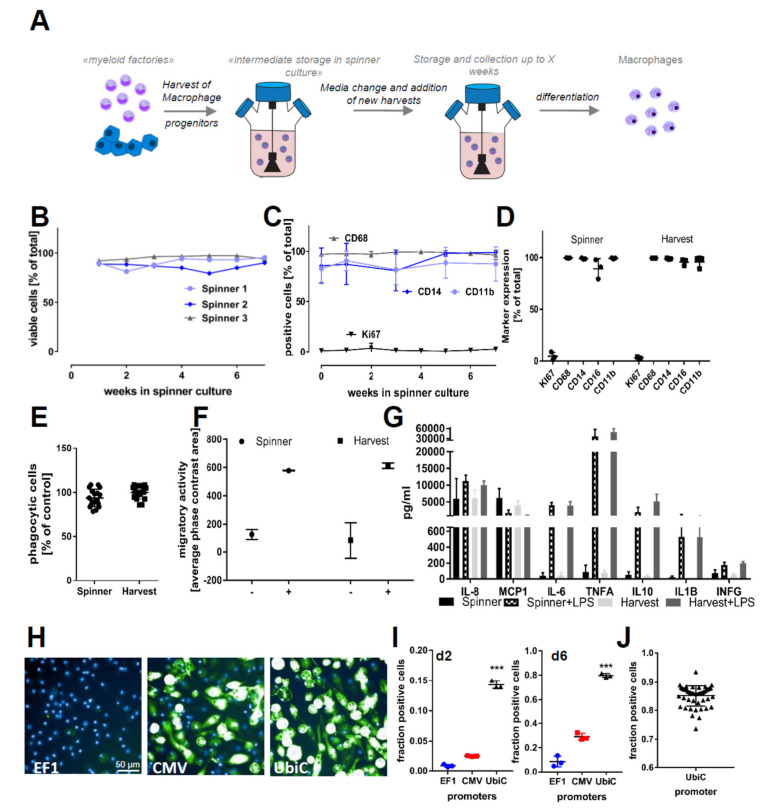
Prolonged cultivation of iPSC–macrophage progenitors and functionality of cells. (**A**) A scheme of prolonged cultivation of macrophage progenitors in suspension culture: the suspension culture allows the accumulation of several harvests over a period of weeks and then the start of macrophage differentiation from a large homogenous population at once. (**B**) Viability of cells in different suspension cultures over the period of 6 weeks: Viability was assessed by analyzing Pi negative cells in flow cytometry. The tested iPSC lines were SFC840-03-01 (STBCi026-B), SFC831-03-03 (STBCi024-B), and SBNeo1. (**C**) Myeloid marker genes (CD14, CD11b, and CD68) and the proliferation marker (Ki67) of monocytes sampled over a period of 6 weeks from suspension cultures: Data are means ± SD (three independent experiments, tested iPSC lines SFC840-03-01 (STBCi026-B), SFC831-03-03 (STBCi024-B), and SBNeo1). (**D**) Myeloid marker genes (CD14, CD16, CD11b, and CD68) and the proliferation marker (Ki67) in cells differentiated from suspension culture and direct harvests. Data are means ± SD (three independent experiments, tested iPSC lines SFC840-03-01 (STBCi026-B), SFC831-03-03 (STBCi024-B), and SBNeo1). Marker expression between suspension culture and direct harvests was tested for statistical significance by one-way ANOVA with Dunnet‘s post hoc test. No significance between the two culture conditions was identified. (**E**) Phagocytic properties of cells derived from suspension storage and directly differentiated after harvesting: Cells were incubated for 2 h with pHrodo-labeled Zymosan and H-33342 and subsequently analyzed by high-content imaging. Phagocytosis was normalized to the percent positive cells of cells differentiated directly from harvests. Data are means ± SD (in three independent experiments, macrophage progenitors and macrophages were derived from Bioneer C10 (H266 C10 GC)). (**F**) Migration capability of cells derived from suspension storage and directly differentiated after harvesting was assessed using the Incucyte transwell assay. Cells were seeded on top of the membrane, and migration to the bottom side of the membrane in the presence or absence of chemoattractant (C5a) in the lower compartment was assessed using the Incucyte migration tool quantifying the occupied phase contrast area on the bottom of the membrane after 60 h of incubation. Data are means ± SD (three independent experiments). (**G**) Cytokine release of cells derived from suspension storage and directly differentiated after harvesting in unstimulated state and stimulated with 100 ng/mL lipopolysaccharide (LPS) for 18 h was assessed. Data are means ± SD (in three independent experiments, macrophage progenitors and macrophages were derived from Bioneer C10 (H266 C10 GC)). (**H**) Representative images of green fluorescent protein (GFP)-positive cells after adenovirus infection: Cells were infected with adenovirus carrying GFP with either the Human elongation factor-1 alpha (EF1), cytomegalovirus (CMV), or ubiquitin C (UBIC) promotor and differentiated for 6 days in 96-well plates. Cells were differentiated from iPSC line SFC831-03-03 (STBCi024-B). (**I**) Quantification of GFP-positive macrophages at d2 and d6 after infection: Data are means ± SEM (three independent experiments). Statistical significance was determined by one-way ANOVA with Bonferroni‘s post hoc test. *** *p* < 0.001. (**J**) To test for scalability, suspension culture was bulk transfected with adenovirus and incubated for 7 days in suspension; then, cells were differentiated for 5 days to M0 macrophages, and the proportion of GFP-positive cells was analyzed using high-content analysis. Data points indicate independent macrophage differentiations from a single suspension culture (*n* = 48). Cells were differentiated from iPSC line SFC831-03-03 (STBCi024-B).

To differentiate iPSC-derived macrophages, we adopted and modified a previously published protocol [31]. Starting from the pluripotent state, we induced mesoderm and subsequent hemogenic endothelium differentiation in preformed and size-controlled embryoid bodies (EBs) for four days by supplementing the culture medium with recombinant human bone morphogenetic protein 4 (BMP4), vascular endothelial growth factor (VEGF), and stem cell factor (SCF). On day 5, floating EBs were reseeded for attachment to culture vessels in “factory” medium containing human macrophage colony-stimulating factor (M-CSF) and interleukin 3 (IL-3) (Figure 1A). Key modifications to the previously published protocol [31] are the maintenance culture of iPSC on Laminin-521-coated plates and the reseeding of the EBs on growth factor-reduced (GFR) Matrigel-coated culture vessels (Figure 1B). These adaptations led to an earlier presence of macrophage progenitors, with substantially increased yields of macrophage progenitors per harvest throughout the differentiation (Figure S2). The strong adherence of EBs to the culture vessel (Figure 1B) resulted in a reduced number of detached EBs. This increased the overall robustness and scalability of the protocol. Myeloid marker expression (CD14, CD11b, and CD68 in the macrophage progenitors (Figure 1C) as well as (CD68, IBA1, CD14 and CD11b) in the terminally differentiated cells) confirmed target cell identity. Respective marker expressions ≥ 90% were monitored throughout the production phase for up to 120 days (Figure 1D). Our modified protocol increased the yield by a factor of 5–7 fold compared to the original protocol from Wilgenburg and colleagues (Figure 1E). The modifications to this protocol allowed us to scale the 2D culture phase to 1000 cm^2^. This culture area already allows a series of 18–25 harvests with single harvest yields of up to 6 × 10^8^ cells. However, for drug development and screening, larger uniform batches are highly desirable. Cryopreservation efforts of both iPSC-derived macrophage progenitors and macrophages resulted in low viabilities. To overcome this limitation, we established a method for prolonged cultivation of macrophage progenitors in suspension, namely “Spinner” cultures (Figure 2A). Harvested macrophage progenitors were transferred to spinner cultures. This enabled the accumulation of several harvests (up to 8 weeks = 16 harvests) from myeloid factories and the storage of macrophage progenitors for up to two months (up to 16 weeks in total: 8 weeks’ accumulation + 8 weeks’ maintenance). The preservation of cellular identity and properties was supported by cell viability scores above 95% (Figure 2B) and macrophage progenitor marker gene expressions of CD68, CD11b, and CD14 (Figure 2C) over the entire spinner culture period (data shown for 6 weeks’ maintenance). We did not observe significant differences in the expression of these markers between M0 macrophages directly differentiated from freshly harvested macrophage progenitors and macrophages derived from macrophage progenitor cultures in spinner cultures (Figure 2D). Furthermore, we assessed functional characteristics of M0 macrophages derived from fresh harvests and of macrophages derived from spinner cultures. Similarly to the myeloid marker expression pattern, macrophages from both conditions exhibited highly similar and reproducible phagocytic capacity, chemotaxis, and cytokine release (Figure 2E–G).

Forced overexpression of genes of interest or modulation of drug target genes is an essential method for biological research and drug discovery to study cellular and molecular function. As primary macrophages are difficult to manipulate genetically, we sought to test adenoviral transduction efficiencies in iPSC-derived myeloid progenitors. For this, we made use of commercial adenoviruses carrying GFP (green fluorescent protein) under the control of three different promoters (CMV, EF1, and UBIC). As readout, we used and quantified the percentage of reporter gene-expressing M0 macrophages 2 and 6 days after infection using the different viral vectors (Figure 2H,I). To further explore the applicability of the spinner cultures, we inoculated myeloid progenitors with virus directly in the spinner, achieving 85.11 ± 3.6% infection efficiency (Figure 2J). This bulk transfection was tested for up to 10^9^ cells at a density of 1.5–2 × 10^6^ cells/mL at once and was highly efficient irrespective of the spinner culture volume (50–500 mL).

### 2.2. iPSC-Derived Macrophages Polarize Comparably to Primary Macrophages

**Figure 3:**
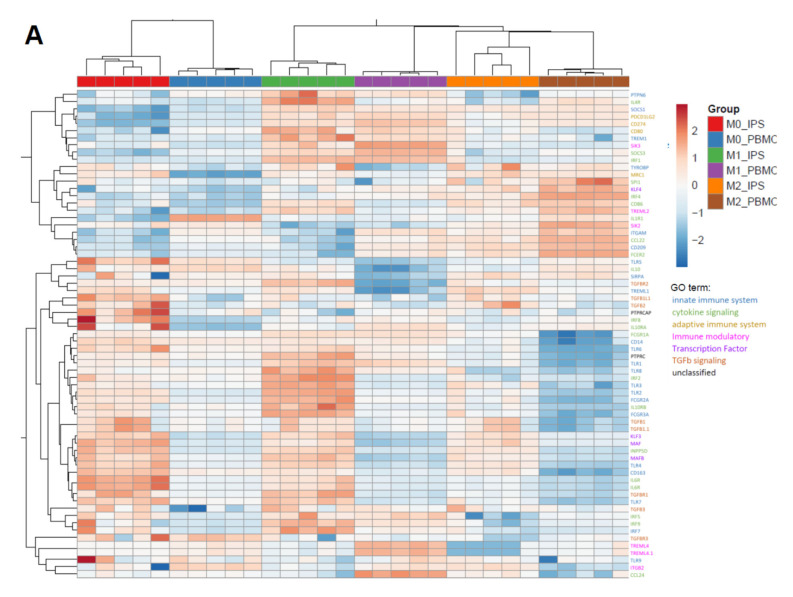
Marker expression comparison of iPSC-derived and peripheral blood mononuclear cells (PBMC)-derived CD14+ cells: (**A**) Heatmap comparing the differential expression of genes characteristic for macrophage polarization. Expression values are depicted as z scores. Red boxes indicate strongest expression among the different groups for each individual gene, and dark blue indicates lowest. Heatmap was generated using ClustVis [32]. iPSC-derived macrophage progenitors and macrophages were derived from SFC840-03-01 (STBCi026-B) and compared to commercial PBMC cells from a single donor. (**B**) Flow cytometry results of myeloid marker Programmed death-ligand 1 (PD-L1) and CD14 of cells derived from either PBMC or iPSC and polarized as described in detail above. Data are means ± SEM (three independent experiments, iPSC-derived macrophage progenitors and macrophages were derived from SFC840-03-01 (STBCi026-B) and compared to cells derived from PBMC from different donors). Statistical significance was determined by two-way ANOVA with Tukey‘s post hoc test. *** *p* < 0.001. (**C**) Refractive index images of macrophages derived from iPSC (SBNeo1) or PBMC and polarized either in M1, M0, or M2. Images were acquired using Nanolive 3D cell explorer. Scale bar represents 20 µm.

**Figure 4:**
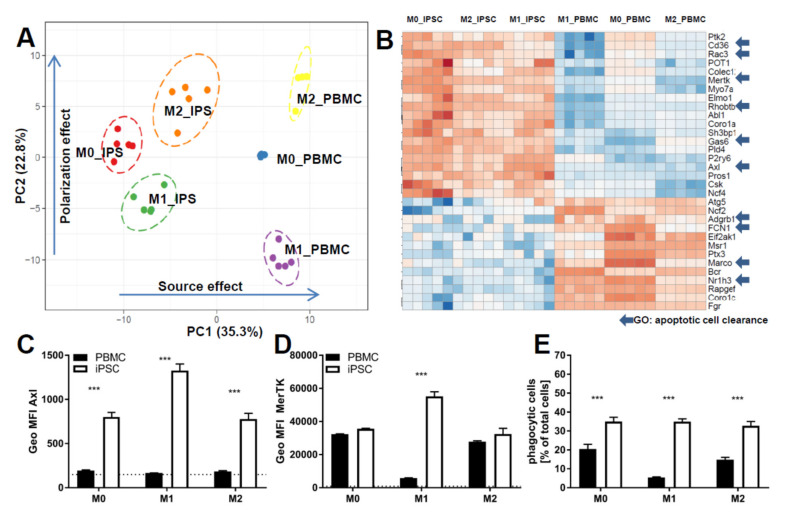
Comparison of functionality of iPSC-derived and CD14+ cells derived from PBMCS: (**A**) Principal component analysis of the expression of the genes of the GO term phagocytosis in cells derived from either iPSC or PBMC and polarized into M0, M1, and M2. Principal component analysis (PCA) was generated using ClustVis [32]. iPSC-derived macrophage progenitors and macrophages were derived from SFC840-03-01 (STBCi026-B) and compared to commercial PBMC cells from a single donor. (**B**) Heatmap of the genes of the GO term phagocytosis in cells derived from either iPSC or PBMC and polarized into M0, M1, and M2: Expression values are depicted as z-factors. Red boxes indicate the strongest expression among the different groups for each individual gene, and dark blue indicates the lowest. Genes belonging to the gene ontology “apoptotic cell clearance” are highlighted by an arrow. Heatmap was generated using ClustVis [32]. iPSC-derived macrophage progenitors and macrophages were derived from SFC840-03-01 (STBCi026-B) and compared to commercial PBMC cells from a single donor. (**C**) Flow cytometry analysis represented as percent positive cells for phagocytosis receptors (AXL and MERTK) of cells derived from either PBMC or iPSC and polarized as described in detail above. iPSC-derived macrophage progenitors and macrophages were derived from SFC840-03-01 (STBCi026-B) and compared to cells derived from PBMC from different donors. (**D**) Mean fluorescence intensity for phagocytosis receptors (AXL and MERTK) of cells were derived from either PBMC or iPSC and polarized as described in detail above. iPSC-derived macrophage progenitors and macrophages were derived from SFC840-03-01 (STBCi026-B) and compared to cells derived from PBMC from different donors. (**E**) Percentage of cells that are positive for efferocytosis of apoptotic Jurkat cells: cells derived from either PBMC (different donors) or iPSC (SFC840-03-01 (STBCi026-B); SBNeo1) and polarized as described in detail above were incubated with pHrodo-labeled early apoptotic Jurkat cells, and efferocytosis was assessed by flow cytometer analysis. Data are means ± SEM (three independent experiments). Statistical significance was determined by two-way ANOVA with Tukey‘s post hoc test. *** *p* < 0.001.

One key functional property of macrophages is the response to diverse types of inflammatory and environmental stimuli and the subsequent polarization into either M1- or M2-like macrophages. To compare our cells to primary cells, we used monocytes either isolated from PBMCs or commercial frozen, CD14-positive cells. Cells were polarized for 7 days either with Granulocyte-macrophage colony-stimulating factor (GM-CSF) and Interferon gamma (IFNγ; M1) or M-CSF (M0), alone or in combination with IL-4 (M2) (Figure 3 and Figure 4). To compare cells on the level of gene expression, from each condition (M0, M1, and M2 derived from commercial CD14-positive cells and iPSC respectively), five replicates were analyzed by RNAseq (Figure S3). Principal component analysis showed that each polarization condition separated from each other, as expected. In line with the literature, the “M0” state was closer to the “M2” state for iPSC- and PBMC-derived macrophages, suggesting that M-CSF alone results in M0 iPSC-macrophages acquiring a default “resolving” state (Figure S3). PBMC- and iPSC-derived subsets clustered together for macrophage-specific genes as well as polarization states (Figure 3A), suggesting an overall similar expression pattern with respect to general macrophage signature genes. We identified two gene clusters induced only for either M1 or M2 polarized cells respectively and a large cluster present in M1 and M0 but downregulated in M2 (Figure 3A). These similarities were further underlined by direct comparison of individual markers by flow cytometry analysis, indicating a high degree of overlap between iPSC-derived macrophages and PBMC-derived ones (Figure 3B, Figures S4 and S5A,B). Moreover, Macrophages derived from iPSC and PBMC showed comparable morphology in the different polarization states (Figure 3C). To focus on the functionality of these cells, we investigated the expression patterns of genes related to phagocytosis, integrating approximately 170 genes associated with the gene ontology (GO)-term phagocytosis. We found a clear separation between iPSC- and PBMC-derived macrophages on the principal component 1 (PC1) (Figure 4A). To further analyze the difference between the iPSC-derived and PBMC-derived cells, we chose differentially expressed genes from this PC1, which had a coherent expression pattern in polarization conditions (Figure 4B). Ten out of the 30 identified genes are known to be involved in the removal process of apoptotic cells, also termed efferocytosis. In concordance with the expression data, we observed significant differences in the frequency of AXL- and MERTK-positive cells as well as in the respective expression levels by flow cytometry (Figure 4C,D and Figure S5C,D). To determine whether these differences would have functional consequences, we performed an in vitro efferocytosis assay. To this end, iPSC- and PBMC-derived macrophages were incubated with live or early apoptotic Jurkat cells and analyzed for efferocytosis by flow cytometry (Figure 4E). The iPSC-derived cells of all polarization stages displayed a higher efferocytotic capacity when compared to their PBMC-derived equivalents. These results are in line with the lower expression levels of AXL and MERTK in PBMC-derived macrophages, especially when polarized towards the M1 phenotype.

### 2.3. The Use of iPSC-Derived Macrophages to Identify Modulators of Phagocytosis and Cytokine Release

**Figure 5:**
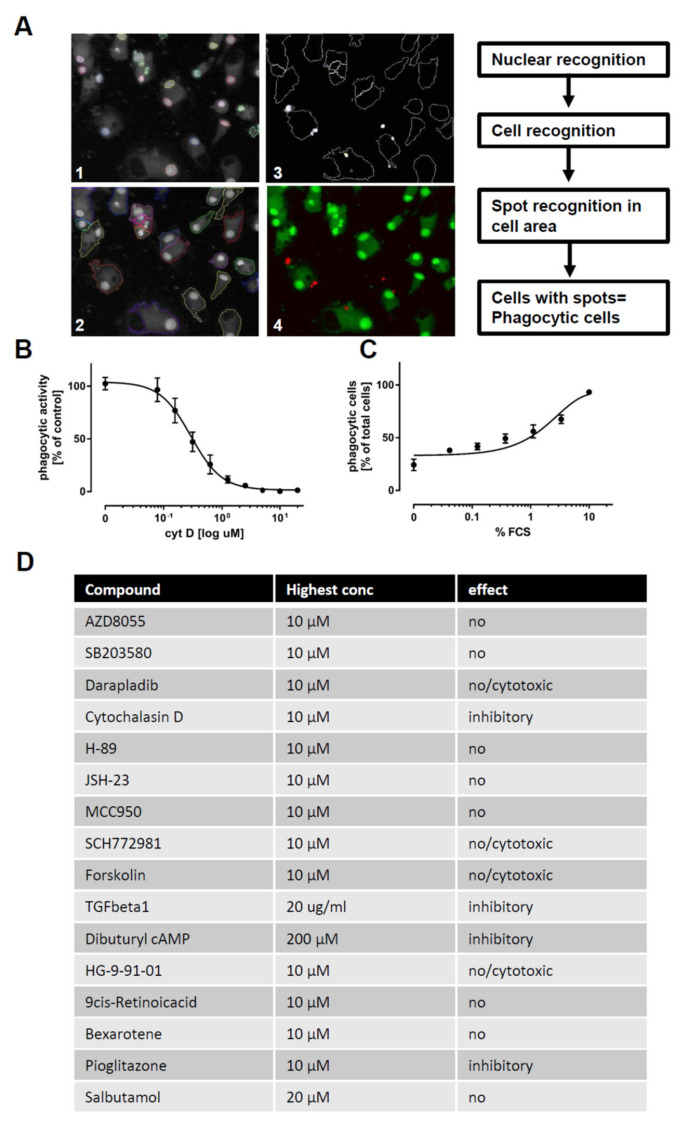
Modulators of phagocytosis: (**A**) Images of macrophages (M0) phagocytosing pHrhodo-labeled Zymosan (orange). Macrophages were stained using viability dye calcein-AM (green) and H-33342 (blue) for schematic depiction of quantification workflow. (**B**) Dose-response curve of the inhibitory effect of cytochalasin D on phagocytosis: Values are normalized to the activity in the vehicle control. Phagocytosis was assessed using automated microscopy (Operetta, Perkin Elmer) and image evaluation (Harmony, Perkin Elmer) (*n* = 3). (**C**) Dose-dependent increase in phagocytosis, mediated by bovine serum opsonization: Depicted as percentage of phagocytic cells relative to total cell count. Phagocytosis was assessed using automated microscopy (Operetta, Perkin Elmer) and image evaluation (Harmony, Perkin Elmer) (*n* = 3). (**D**) Tabular summary of the 16 tested tool compounds: Compound name, highest used concentration, and outcome classification are indicated in the table. Phagocytosis was assessed using automated microscopy (Operetta, Perkin Elmer) and image evaluation (Harmony, Perkin Elmer). iPSC-derived macrophage progenitors and macrophages were derived from SFC840-03-01 (STBCi026-B).

**Figure 6:**
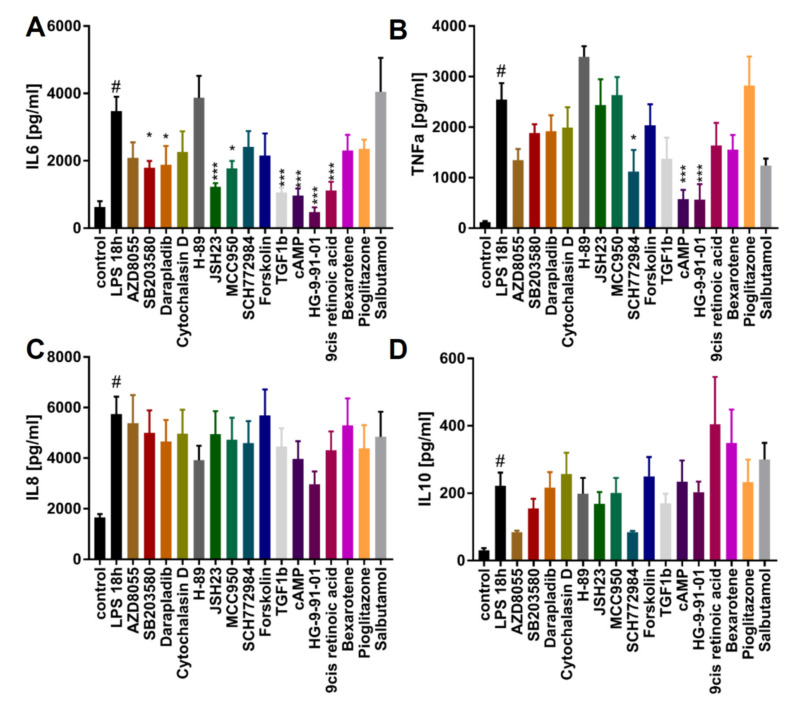
Identifying modulators of cytokine release: The modulatory effect of tool compounds on cytokine secretion by macrophages upon LPS stimulation. Cells were pretreated with the highest concentration indicated in Figure 5F of the respective compound for 1 h and then stimulated for 18 h with 100 ng/mL LPS. After 18 h, supernatant was collected and Interleukin-6 (IL-6), Tumor necrosis factor alpha (TNFα), Interleukin-8 (IL-8), and Interleukin-10 (IL-10) levels were determined (*n* = 3). (**A**) Effect of compounds on IL-6. (**B**) Effect of compounds on TNFα. (**C**) Effect of compounds on IL-8. (**D**) Effect of compounds on IL-10. Data are means ± SEM (three independent experiments). Statistical significance was determined by one-way ANOVA with Dunnet‘s post hoc test in comparison to LPS treatment. iPSC-derived macrophage progenitors and macrophages were derived from SFC840-03-01 (STBCi026-B). *** *p* < 0.001; ** *p* < 0.005; * *p* < 0.05.(compared to LPS stimulated); # *p* < 0.05 (compared to unstimulated).

Having established the differentiation protocol and confirmed an overall macrophage expression profile by RNAseq and flow cytometry, we next assessed whether the iPSC-derived macrophages faithfully recapitulate macrophage function. For this, we chose 16 compounds reported to modulate targets that are either affected by changes in cyclic adenosine monophosphate (cAMP) [33,34,35,36,37,38,39,40] or to have immune modulatory effects [41,42,43,44,45,46] (Figure S6). These compounds were screened for modulatory effects on the phagocytosis of Zymosan particles (Figure 5) and on cytokine release (Figure 6) to demonstrate the applicability of iPSC-derived macrophages in small molecule screens with functional endpoints.

In order to assess the phagocytic activity of macrophages, we differentiated the cells for 6 days using M-CSF. Afterwards, cells were exposed to different compounds for 24 h, and pHrodo-labeled Zymosan was added for the last two hours. Cells were fixed, and phagocytosis was assessed using high-content imaging. As a control, phagocytic capacity was decreased by pretreatment with cytochalasin D to inhibit actin polymerization or was augmented by fetal calf serum (FCS) pretreatment to opsonize zymosan and to facilitate its uptake (Figure 5A–D). In order to exclude false positive hits, which affect macrophage number by cytotoxic side effects, we also monitored the number of macrophages per well. Using the assay, we found cytochalasin D, TGFβ-1, cAMP, and pioglitazone to exhibit inhibitory effects on phagocytosis, while the other compounds had no effect or displayed cytotoxicity at higher concentrations (Figure 5E and Figure S7). None of the compounds increased the phagocytosis of zymosan. 

Yet another immune regulatory function of macrophages is to release pro- or anti-inflammatory cytokines upon stimulation, which makes them an attractive target in drug discovery. To illustrate this, we pretreated the M0 macrophages with the 16 compounds for 1 h, followed by stimulation with LPS for 18 h (Figure 6A–D). LPS exposure triggered significant elevations of IL-6, TNFα, IL-8, and IL-10 release. We found AZD8055, SB203580, darapladib, JSH23, MCC950, TGFβ-1, cAMP, HG9-91-01, and 9-cis-retinoic acid to significantly decrease IL-6 release in response to LPS (Figure 5A). Moreover, SCH772984, cAMP, and HG9-91-01 significantly inhibited the release of TNFα (Figure 5B). None of the tested compounds had significant effects on IL-8 or IL-10 secretion. These results underline the functional responsiveness of iPSC-derived macrophages in terms of cytokine response to pro-inflammatory triggers and implementation in screening assays.

### 2.4. The Use of iPSC-Derived Macrophages to Screen for Modulators of Calcium Release and Chemotaxis

**Figure 7:**
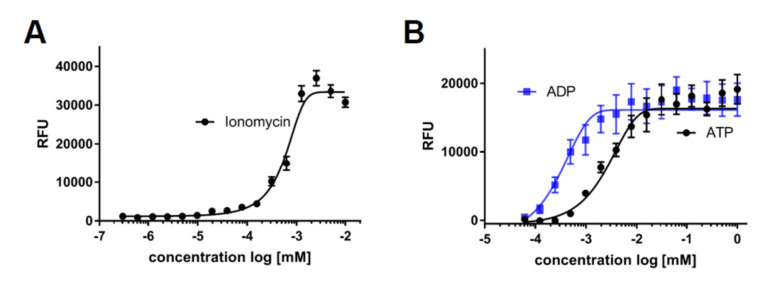

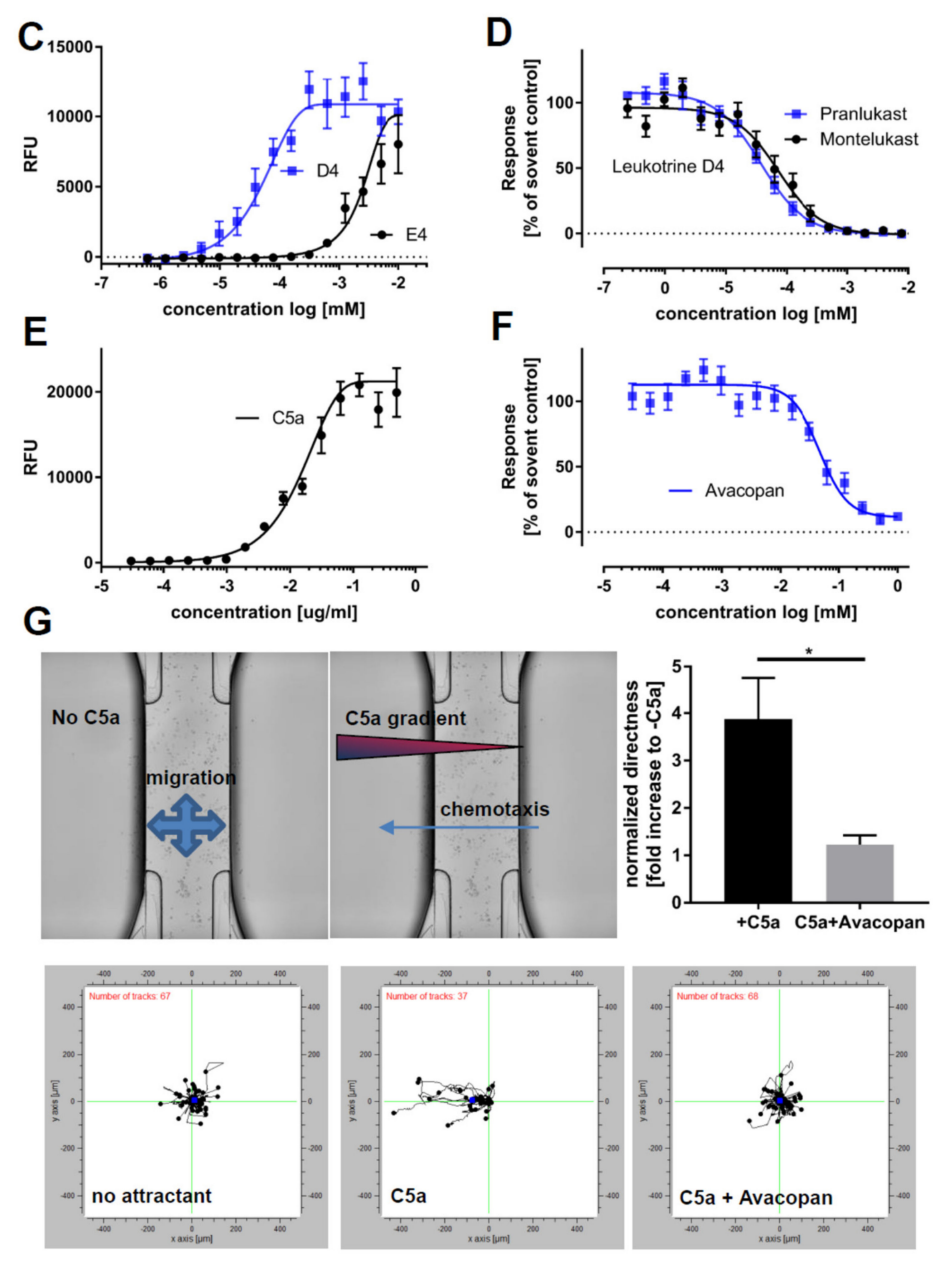
Stimulators of calcium release: Intracellular calcium kinetics following different stimuli. Intracellular calcium was assessed using the Fluoro-6 Kit (Molecular Devices) and the FDSS7000 Functional Drug Screening System (Hamamatsu). (**A**) Intracellular calcium levels upon cell permeabilization with different concentrations of ionomycin: Maximum measured fluorescence is indicated as relative fluorescence units (RFU), which are baseline corrected (*n* = 3). (**B**) Intracellular calcium levels upon treatment of different concentrations of ADP or ATP: Maximum measured fluorescence is indicated as relative fluorescence units (RFU), which are baseline corrected (*n* = 3). (**C**) Intracellular calcium levels upon treatment of different concentrations of leukotrienes D4 or E4: Maximum measured fluorescence is indicated as relative fluorescence units (RFU), which are baseline corrected (*n* = 3). (**D**) Dose-dependent inhibition of calcium release by pranlukast or montelukast following leukotriene D4 exposure: Cells were pretreated for 20 min with indicated concentrations of pranlukast or montelukast, and calcium release was then stimulated by leukotriene D4 at the EC80 concentration calculated from 7C (*n* = 3). (**E**) Intracellular calcium levels measured after exposure to different concentrations of complement component 5a (C5a). Maximum measured fluorescence is indicated as relative fluorescence units (RFU), which are baseline corrected. (**F**) Dose-dependent inhibition of calcium release by avacopan: Cells were pretreated for 20 min with indicated concentrations of avacopan, and calcium release was then stimulated by C5a at the EC80 concentration calculated from 7E. (**G**) Directed migration to the chemoattractant C5a was assessed using ibidi^®^ chemotaxis slides. The vehicle was treated to monitor baseline movement, C5a was treated in the left compartment to assess directed migratory behavior, and C5a was treated in the left compartment in the presence of avacopan to test for C5a receptor specificity. Representative single cell tracking blots of one experiment are shown, and values for the directness were normalized to the condition without C5a (*n* = 3). iPSC-derived macrophage progenitors and macrophages were derived from SFC840-03-01 (STBCi026-B), SBNeo1, and SBAD3-01.3.* *p* < 0.05.

A further key functionality of macrophages is to sense damage and to migrate to the affected area. This sensing is in large part mediated by ligand binding to their cognate receptors, followed by calcium release and subsequent directed chemotaxis. In order to analyze this cellular process, we monitored calcium flux in response to various stimuli. As a threshold, we determined maximal calcium response of iPSC-derived macrophages using the ionophore ionomycin (Figure 7A) and assessed the response to common damage signals or inflammatory mediators by exposure to ATP, ADP, and leukotriene D4 and E4 (Figure 7B,C). To validate ligand-specific effects of stimulation, we preincubated the cells with the known leukotriene receptor antagonists pranlukast and montelukast, which both dose-dependently blocked the calcium release triggered by leukotriene D4 (Figure 7D).

## 3. Discussion

The anaphylatoxin C5a, as part of the complement system, is a well-described chemoattractant for innate immune cells. We found macrophages to react with an increase in intracellular calcium following C5a exposure (Figure 6E). This effect was dose-dependently inhibited using the C5a receptor antagonist avacopan (Figure 6F). In order to assess whether the exposure to C5a would induce chemotactic migration, we monitored the movement of cells in a gradient of C5a in the presence or absence of the C5a receptor antagonist avacopan. C5a induced directed migration towards the higher concentration of the gradient, which was inhibited by avacopan. To connect calcium release with a functional cellular readout, we tested the chemotaxis of M0 macrophages towards a gradient of C5a using an ibidi^®^ chemotaxis assay and using the EC50 concentration of C5a as previously determined by calcium response. The chemotaxis assay tracks the movement of single cells and measures speed and direction, enabling to distinguish between chemotaxis and chemokinesis. In this assay, macrophages showed directed migration towards the C5a gradient, which could be inhibited by preincubation with the C5a receptor antagonist avacopan (Figure 7G and Figure S8 (videos)). 

Our results demonstrate the functionality of the macrophages and the feasibility of this in vitro model system to address calcium release in macrophages in response to different stimuli and compounds. Moreover, we could correlate a primary assay (calcium release) with a functional endpoint in a secondary assay (directed migration).

Myeloid cells are a major component of the innate immune system, and dysregulation of their pleiotropic function is associated with the initiation and progression of numerous diseases [1,5]. Research in this field faces many challenges: among others, donor variability of primary blood-derived cells, difficult isolation of tissue-resident macrophages, and insufficient cell numbers for screening compromise current drug discovery projects. One frequently used approach to overcome some of these limitations is the use neoplastic or immortalized cell lines such as THP-1. However, these cells are continuously dividing or can only be differentiated by administration of chemicals. Furthermore, they have a limited capacity to resemble the in vivo situation [11,12,13]. Here, we highlight how these limiting factors may be overcome by upscaling and improving an iPSC-based differentiation protocol resembling myb-independent myelopoiesis of iPSC-derived macrophages. Compared to primary blood-derived macrophages, they display similar polarization patterns. However, in terms of functionality and polarization, they showed a higher efferocytosis rate and a bias towards M2 type macrophage polarization. Cells derived via this protocol resemble macrophages of primitive hematopoiesis and are therefore ideal precursors for the generation of macrophages resembling key features of tissue-resident macrophages such as alveolar macrophages or microglia cells [18,19,28,30]. Until very recently, especially microglia research was almost exclusively based on primary cells isolated from rodents. This has fundamentally changed with the advent of stem cell-based protocols. We and others have already demonstrated the feasibility and applicability of iPSC-derived myeloid cells for the generation and study of microglia-like cells in 2D and 3D cultures [9,18,47,48]. Thus, these methods will improve macrophage research and drug discovery while reducing the need for animals for the isolation of primary cells.

The improvements described in this study have a great potential for culture scalability as outlined above. The limited bioavailability of human primary cells can be overcome by the use of the macrophage-like cells described here. The improved method enabled us to harvest for a longer period (up to 120 days) with a higher yield (up to 7×) compared to Wilgenburg et al. [31]. We managed a robust culture of myeloid factories in culture vessels with up to 1000 cm^2^, resulting in 20–60 million macrophage progenitors per harvest. Recently published work of others also aimed at increasing the yield or at decreasing the differentiation time of similar protocols [19,49]. It was shown that the myeloid factory state can be transferred to stirring cultures, which could be run in industry compatible bioreactors to generate sufficient cells for cell therapy approaches [19]. The authors of this study report harvests of 1–3 × 10^7^ cells/week, which is factors of 2–4 below the harvests we report here, with the same amount of culture media. While stirring cultures have clear advantages in scalability, harvests from such cultures by sedimentation, filtration, and centrifugation are more laborious. A different study was successful in shortening the differentiation to 15 days [49] and in generating about 40 macrophage progenitors per iPSC in a single harvest differentiation. However, in order to obtain the macrophage progenitors, the study had to use a CD14 immunoferromagnetic purification step, which is not required for the method described here. Since all of the here described methods have their specific advantages, the combination thereof should be addressed in the future. This could lead to an even faster and more robust large-scale myeloid differentiation method.

Several studies already showed that the generation of iPSC-derived macrophage progenitors and cells differentiated thereof are a valuable source of patient specific cells and can be used to model and understand disease underpinning mechanisms [9,19,48,50,51,52]. Moreover, they are a powerful tool to dissect which properties are intrinsic to all macrophages and which are due to ontogenetic differences or tissue residency [8]. One example for such difference is the efferocytotic capacity, which has been shown to be different between iPSC- and PBMC-derived macrophages [49]. This is in concordance with our findings reported here.

The number of cells generated with one of the aforementioned protocols is already sufficient to stably supply profiling activities in functional cellular assays. However, small to medium-sized small molecule screens based on phenotypic differences or genetic screens require more cells.

Since the derivation of target cells from iPSCs is rather lengthy, an accumulation of macrophage progenitors from multiple harvests is required to supply several drug discovery processes. Cryopreservation of intermediate stages, in this case macrophage progenitors, would be highly desirable. While others reported progress in this area [49], we have not managed to cryopreserve cells with good recovery rates, which will be a point to further address and optimize in the near future. However, by introducing and characterizing the prolonged cultivation of macrophage progenitors in suspension culture, we were able to overcome this limitation by various means. Combining the first protocol improvement with the suspension culture, we were able to accumulate up to 1.5 × 10^9^ cells from one production period of a myeloid factory. Given the available culture dishes (up to 10,000 cm^2^) and the suspension accumulation, this protocol has even more scalability potential (at least by a factor of 10) and could allow for single concentration screens of compounds in the 6–7-digit range.

Bulk transfection of suspension cultures of unprecedented scale at infection rates of up to 90% underlies the applicability for genome-wide screening to elucidate gene function. The method represents a valuable tool for the use of pluripotent stem cells in disease modeling, target validation, and next-generation phenotypic screening where the availability of large numbers of defined and functional cell types is essential. This and other approaches of deriving macrophages at scales relevant for drug discovery could possibly be leveraged for cell therapy [19] where, apart from cellular authenticity, bioprocess robustness and scalability are prerequisites.

## 4. Materials and Methods 

### 4.1. iPSC Maintenance

Human iPSC lines used for protocol optimization were SFC840-03-01 (STBCi026-B) [53], SFC831-03-03 (STBCi024-B) [9] (both generated in the StemBANCC consortia and deposited at EBiSC), SBNeo1, SBAD3-01 [54] (all reprogrammed with Life Technologies Cytotune Sendai virus), and Bioneer C10 (H266 C10 GC) (reprogrammed using an episomal system). Experiments were carried out with available cells from ongoing differentiations. The origin of the cells used for the different experiments is indicated in the figure legends. iPSCs were cultured according to Wilgenburg et al. [31], except the culture dishes (Corning, Somerville, MA, USA) were coated with 12.5 µg/mL rhLaminin-521 (BioLamina, Sundbyberg, Sweden) in PBS containing calcium and magnesium for at least 2 h prior to use. Human iPSCs were seeded and cultured in mTesR1 medium (StemCell Technologies, Vancouver, BC, Canada) at 37 °C with 5% CO_2_, and the medium was changed daily. Cells were passaged at 90% confluence; when the medium was removed, the cells were washed once with PBS and detached with Accutase for 2 to 5 min at 37 °C. After removal of Accutase by centrifugation, the cells were either used for maintenance or the start of differentiation.

### 4.2. Embryoid Body Generation

This step was performed as previously described by Wilgenburg et al. [31]. Briefly, to obtain uniformed EBs, iPSCs were plated into AggreWell 800 (StemCell Technologies, Vancouver, BC, Canada) plates. Two milliliters of mTesR1, supplemented with 10 μM ROCK inhibitor (Y27632, Calbiochem/ Millipore, Burlington, MA, USA) and containing a single cell suspension of 4 × 10^6^ single iPSCs, was added to each AggreWell and centrifuged for 3 min at 100 g to ensure an even and fast distribution of the iPSCs to the AggreWell microwells. The next day, mesoderm induction and subsequent hemogenic endothelium induction were started by exchanging 75% (replacing twice 1 mL of the 2 mL in each well) of the mTeSR1 medium with fresh mTeSR1 medium supplemented with 50 ng/mL human Bone morphogenetic protein 4 (hBMP4) (R&D Systems, Minneapolis, Minnesota, USA), 50 ng/mL human vascular endothelial growth factor (hVEGF) (R&D Systems, Minneapolis, Minnesota, USA), and 20 ng/mL human stem cell factor (hSCF) (R&D Systems, Minneapolis, Minnesota, USA) and were repeated the following two days. A complete list of reagents and suppliers is summarized in Supplementary Figure S1.

### 4.3. Plating of EBs and Continued Maturation along the Myeloid Lineage

At day 4 of differentiation, EBs were harvested by gently dislodging the EBs by rinsing the AggreWells with PBS. EBs were collected in a 40-µm strainer and transferred to “factory” medium, consisting of X-VIVO 15 medium (Lonza, Basel, Switzerland) supplemented with 2 mM Glutamax, 1% penicillin/streptomycin, 50 ug/mL mercaptoethanol, M-CSF (100 ng/mL)(Miltenyi Biotech, Bergisch Gladbach, Germany), and IL3 (25 ng/mL) (Miltenyi Biotech, Bergisch Gladbach, Germany). EBs were plated with a density of 1 EBs/cm^2^ on cell culture vessels (2–1000 cm^2^/6-well cell dish). In contrast to the description of Wilgenburg et al. [31], the EBs were plated on dishes pre-coated for 1 h at room temperature (RT) with growth factor reduced Matrigel (Corning, Somerville, MA, USA) diluted in cold DMEM F12 1:1 1× Glutamax (Gibco/Thermo Fisher, Carlsbad, CA, USA). In order to allow adherence of EBs, EBs were evenly distributed by slow movements and culture vessels were placed immediately at 37 °C with 5% CO_2_ without any further disturbance for the first week of differentiation. During the following two weeks of differentiation, 50% of the starting volume of fresh factory medium was added once a week. From the third week of differentiation, half-medium changes were done until the production and release of (CD14+) macrophage progenitors in the supernatant was observed. From this point on, complete medium change with fresh factory medium was performed twice a week.

### 4.4. Macrophage Progenitor Harvesting and Macrophage Differentiation

Macrophage progenitors were collected from the supernatant by centrifugation (4 min, 300 g), cells were resuspended and counted, and quality control (CD68, Ki67, CD11b, and CD14) of marker expressions by flow cytometry was performed weekly. Macrophage progenitors were transferred to differentiation medium and differentiated to macrophages. According to application requirements, macrophages were either directly differentiated in the required plate format or pre-differentiated for 6 days and then replated to the final plate format one day prior to assay start. For differentiation, cells were cultured in X-VIVO 15 (supplemented with 2 mM Glutamax, 1% pen/strep, and 100 ng/mL M-CSF) at a density of 150,000 cells per cm^2^. The medium was changed three days after plating; cells were differentiated for seven days.

### 4.5. Polarization of Macrophages

Macrophage progenitors were either differentiated into unpolarized (M0) macrophages using XIVIVO15 medium supplemented with 2 mM Glutamax, 1% pen/strep, and 100 ng/mL M-CSF or polarized into pro-inflammatory (M1) or regulatory phenotypes (M2). M1 polarization was induced by the addition of GM-CSF and 50 ng/mL IFNγ instead of M-CSF. M2 polarization was induced by the addition of 100 ng/mL M-CSF and 50 ng/mL IL-4. Unless otherwise stated, cells were differentiated for seven days prior to the experiments.

### 4.6. Prolonged Cultivation in Suspension Cultures

Freshly harvested macrophage progenitors were collected and cultured over several weeks in suspension cultures. To this end, disposable spinner flasks (Corning, Somerville, MA, USA) were placed on magnetic stirrers (30 rpm) in a humidified incubator (37 °C, 5% CO_2_). Cells were cultured in X-VIVO 15 medium (Lonza, Basel, Switzerland) supplemented with 2 mM Glutamax, 1% penicillin/streptomycin, 50 ug/mL mercaptoethanol, M-CSF (100 ng/mL), and IL3 (25 ng/mL). To accumulate sufficient cells for screening purposes, several harvests of one “factory” were accumulated in the suspension cultures over the course of several weeks. Cell number was adjusted to 0.5–2 × 10^6^ cells/mL, 50% medium exchange was performed twice a week, cells were resuspended and counted, and quality control was performed by assessing marker gene expressions (CD68, Ki67, CD11b, and CD14) by flow cytometry.

### 4.7. Isolation of Monocytes from PBMC-and Monocyte-Derived Macrophage Differentiation

Buffy coats of healthy human donors who gave their informed consent were obtained from the Center for Blood Transfusion, University Medical Center, Bern, Switzerland. One part of the buffy coat was diluted with an equal amount of sterile PBS without Ca^2+^ and Mg^2+^(-/-). PBMCs were isolated by centrifugation in Leucosep tubes (Greiner, Kremsmünster, Austria) containing Ficoll (Sigma, St. Louis, MO, USA) according to the manufacturer’s protocol. After centrifugation, the leukocyte ring (including monocytes) was collected in a fresh tube and washed twice with sterile PBS. Red blood cell lysis was performed using Pharm Lyse buffer (BD, Franklin Lakes, NJ, USA) according to the manufacturer’s protocol. Cells were counted in trypan blue solution using a Countess cell counter (Invitrogen/Thermo Fisher, Carlsbad, CA, USA). Monocytes were isolated using the EasySep^TM^ human monocyte isolation kit and RoboSep device (StemCell Technologies, Vancouver, BC, Canada). Monocyte-derived macrophages were differentiated as described above.

### 4.8. Culture and Polarization of CD14-Positive Cells

CD14-positive cells (LONZA, Basel, Switzerland) obtained from a single donor were thawed and subsequently treated comparable to the macrophage progenitors obtained from the iPSC differentiations. Briefly, for differentiation, cells were cultured in X-VIVO 15 (supplemented with 2 mM Glutamax, 1% pen/strep, and 100 ng/mL M-CSF) at a density of 150,000 cells per cm^2^. The medium was changed three days after plating; cells were differentiated for seven days. If required, M1 polarization was induced by the addition of GM-CSF and 50 ng/mL IFNγ instead of M-CSF. M2 polarization was induced by addition of 100 ng/mL M-CSF and 50 ng/mL IL-4. Unless otherwise stated, cells were differentiated for seven days prior to the experiments.

### 4.9. Quality Control and Marker Expression by Flow Cytometry

In order to prepare Flourescence- activated cell sorting (FACS) buffer systems, one part PERM/FIX1 solution was diluted with four parts PERM/FIX2 solution to obtain buffer 1, and one part PERM was diluted with 10 parts ddH2O in order to obtain buffer 2. Cell suspension of pre-Mac cultures or detached macrophages were centrifuged (300 g for 4 min at RT) to remove supernatant. Cells were resuspended in autoMACS running buffer or PBS. The cell number was adjusted, and 250,000 cells/well were added to a 96-deep-well Eppendorf plate. 

For the surface staining, 1 µL of each surface antibody (CD14, CD11b, and CD16) and 1 µL of pre-mix solution were added to a deep well and incubated for 10 min at RT in the dark. Cells were washed by the addition of 550 µL/well autoMACS running buffer and subsequent centrifugation (300 g for 4 min at RT). The supernatant was discarded, and cells were resuspended in 300 µL/well freshly prepared cold buffer 1 and incubated for 30 min at 4 °C. After incubation, cells were washed again as described above and resuspended in 100 µl/well freshly prepared cold buffer 2.

For inside staining, 1 µL in each case of CD68 + Ki67 or re-affinity (REA) control + PermFOXP3 staining buffer were mixed and 1.5 µL of pre-mix solution was added to a deep well and incubated for 30 min at 4 °C in the dark. For washing, 500 µL of buffer 2 was added per well, centrifuged (300 g for 4 min at RT), and resuspended in 100 uL/well freshly prepared cold autoMACS running buffer. A complete list of reagents and suppliers is summarized in Supplementary Figure S1.

### 4.10. Comparative Marker Expression by Flow Cytometry

Macrophages were differentiated from iPSC-derived progenitor cells or from monocytes isolated from PBMCs, as described, in X-VIVO 15 medium for seven days. Cells were washed with PBS and detached using Accutase according to the manufacturer’s protocol and transferred to 96-well V-bottom plates. Fc receptors were blocked by incubating cells for 10 min with flow cytometry buffer containing human fc-block (BD, Franklin Lakes, NJ, USA). Cells were then stained using the following antibodies: CD14-Pe (Thermo Fisher, Carlsbad, CA, USA), Axl-APC (R&D Systems, Minneapolis, Minnesota, USA), MerTK-Pe (Biolegend, San Diego, CA, USA), and PD-L1-BV711 (Biolegend, San Diego, CA, USA) for 30 min in the refrigerator. Cells were washed with PBS and acquired on a Cytoflex from Beckman Coulter and analyzed using FlowJo V10. A complete list of reagents and suppliers is summarized in Supplementary Figure S1.

### 4.11. Characterization by RNAseq

iPSCs (SFC840-03-01 (STBCi026-B)) were differentiated into macrophage progenitors, and these were further differentiated and polarized as described above. In parallel, CD14-positive cells obtained from LONZA from a single donor were treated, cultured, and polarized under the same conditions. All cultures were started on five different days to obtain five independent replicates for the RNAseq experiment. After the polarization period, the cells were lyzed and RNA was extracted using RNeasy Kit from Qiagen. A DNase1 digestion step was performed to avoid genomic DNA contamination. RNA purity was assessed using the Agilent 2100 Bioanalyzer. Strand-specific mRNA-seq libraries were generated from 1 µg total RNA using the TruSeq Stranded mRNA library prep kit (Illumina) according to the manufacturer’s instructions. Briefly, mRNA was purified from total RNA by polyA capture, fragmented, and subjected to first-strand cDNA synthesis. The second-strand synthesis was performed incorporating Deoxyuridine Triphosphate dUTP instead of Deoxythymidine triphosphate dTTP to ensure strand-specificity. Barcoded DNA adapters were ligated to both ends of the double-stranded cDNA and subjected to PCR amplification. The resulting libraries were checked on an AATI Fragment Analyzer, quantified with Qubit, and pooled. The resulting library pool was diluted for cluster generation on the cBot2 and finally sequenced on the Illumina HiSeq 4000 platform.

### 4.12. RNAseq Analysis

Base calling was performed with BCL to FASTQ file converter bcl2fastq v2.17.1.14 from Illumina (https://support.illumina.com/downloads.html). In order to estimate gene expression levels, paired-end RNASeq reads were mapped to the human genome (hg38) with STAR aligner version 2.5.2a using default mapping parameters [55]. Aligned reads were quality checked with FastQC and MultiQC version 1.7 [56,57]. Numbers of mapped reads for all RefSeq transcript variants of a gene (counts) were combined into a single value using SAMTOOLS software (Genome research limited, United Kingdom) [58] and normalized as RPKMs (number of mapped reads per kilobase transcript per million sequenced reads [59]). RNA-seq data have been deposited in the Gene Expression Omnibus database (GEO accession number GSE149377).

### 4.13. Phagocytosis Assay

Eight thousand macrophage progenitors per 384 wells were seeded in 20 µL differentiation medium containing 100 ng/mL M-CSF. After 72 h, an additional 20 µL of differentiation medium was added and the cells were differentiated for a further 72 h. Ten microliters of compounds diluted in differentiation medium were added to the cells for 24 h; 0.5 ng Zymosan labeled with pHrodo and 2 µM H-33342 in 10 µL were added to the cells for the last 2 h of compound treatment. After incubation, the cells were washed with cold PBS, fixed in 4% paraformaldehyde (PFA) for 5 min, placed in PBS, and imaged and analyzed with a high-content imager (Operetta/Perkin Elmer, Waltham, MA, USA).

### 4.14. Efferocytosis Assay

Macrophages were differentiated in vitro from iPSC-derived progenitors as described above. In brief, 5 × 10^4^ cells/well were cultured in culture medium (X-VIVO 15, 1% P/S, and 2 mM Glutamax) with indicated stimuli for seven days in 96-well flat-bottom plates (Corning, Somerville, MA, USA). Jurkat cells were obtained via the Roche Non-Clinical Biorepository. Jurkat cells were cultured in RPMI1640 medium supplemented with 2 mM Glutamax (Gibco/Thermo Fisher, Carlsbad, CA, USA) and 10% FBS and MEM nonessential amino acids (Gibco/ Thermo Fisher, Carlsbad, CA, USA). Apoptosis was induced by culturing cells at 1 × 10^6^ cells/mL medium containing 2.5 µM staurosporine (Sigma, St. Louis, MO, USA) at 37 °C at 5% CO_2_ for three hours. Afterwards, cells were labeled with pHrodo Red (Invitrogen/Thermo Fisher, Carlsbad, CA, USA) according to the manufacturer’s protocol and washed twice with PBS, 1% bovine serum albumin (BSA), and 1 mM Ethylenediaminetetraacetic acid (EDTA) and once with PBS. Apoptosis induction was verified by flow cytometry using AnnexinV-Fitc staining kit (BD, Franklin Lakes, NJ, USA) with fixable viability dye APCeF780 (eBioscience/ Thermo Fisher, Carlsbad, CA, USA). Apoptotic cells were added to macrophages at a ratio of 6:1 (Jurkat:macrophages) in culture medium for two hours. Cell were washed once with PBS, detached with Accutase, and analyzed for pHrodo fluorescence using flow cytometry.

### 4.15. Cytokine Release

Cells were differentiated and polarized as described above. The medium was exchanged prior to the experiment, and culture volume reduced to 50% to increase potential signals. Cells were pretreated for 1 h with test compounds, followed by stimulation with 100 ng/mL LPS to trigger cytokine release. Supernatants were collected after 18 h and analyzed using FirePlex-HT 1 (Abcam, Cambridge, United Kingdom), detecting IL-2, -4, -6, -8, -10, and -17A; MCP-1; IFNγ; TNF; α and IL-1β. Cytokine analysis was performed according to the manufacturer’s instructions. Briefly, samples were diluted 1:1 in sample dilution buffer, and 12.5 µL was transferred to the assay plate. Antibody mix and particle mix were diluted and mixed, and 12.5 µL per well was added to the assay plate using a multidrop system (Thermo Fisher, Carlsbad, CA, USA). Plates were sealed and incubated overnight on an orbital shaker (1200 rpm, RT). After incubation, 10 µL of imaging dye was added to each well of the assay plate using a multidrop system (Thermo Fisher, Carlsbad, CA, USA). Plates were sealed and incubated for 20 min on an orbital shaker (1200 rpm, RT). Images of the antibody-coated fluorescent particles were acquired using an Operetta CLS (Perkin Elmer, Waltham, MA, USA) with a 5× objective and the channel settings as listed in the manufacturer’s protocol. Pictures were analyzed using FirePlex Analysis Workbench (Abcam, Cambridge, United Kingdom). Absolute values were calculated using an 8-point 1:3-diluted standard curve.

### 4.16. Transwell Migration

Cells were differentiated as described above (7 days M0), detached from the dishes with Accutase, and plated at a density of 8000 cells per well in a 96-well IncuCyte ClearView cell migration plate. In the lower compartment, either recombinant human C5a (4 ng/mL) or solvent control were added as chemoattractant. Plates were incubated in an IncuCyte S3, and images were acquired using the 10× objective every 4 h for upper and lower wells. Migration was assessed for 72 h and quantified using the IncuCyte software migration analysis tool.

### 4.17. Chemotaxis

Cells were differentiated as described above (7 days M0), detached from the dishes with Accutase, and plated onto the ibidi^®^ µ-Slide Chemotaxis. C5a was applied to either one, both, or none of the compartments to assess directed migration (chemotaxis). To block C5a-mediated chemotaxis, cells were pretreated for 5 min with 500 nM C5a receptor antagonist avacopan. Transmitted light pictures were acquired every 20 min using the PICO automated imager (Molecular Devices, San Jose, CA, USA) with a 10× objective. Pictures were exported as Tagged Image File Format(TIFF) and analyzed using the ImageJ plug-in provided by ibidi^®^.

### 4.18. Calcium Assay

Cells were differentiated as described above, and 12,000 macrophage progenitors were plated per well of a 384-well plate and were differentiated to M0-like macrophages for 5 days (higher cell numbers produced more consistent results in the well intensity readout of the Fluorescent Imaging Plate Reader (FLIPR) reader). For calcium measurements, cells were incubated with the FLIPR calcium 6 imaging dye (Molecular Devices, San Jose, CA, USA) following the manufacturer’s instructions. Briefly, dye was dissolved in 10 mL of assay buffer 1, and 20 µL per well was added to the cells. Cells were incubated for 2 h with the dye. Calcium release to ATP, ADP, leukotrienes D4 and E4, as well as C5a was assessed using the Hamamatsu FDSS7000 detection system. Background signal was assessed by 10 pictures prior to the addition of stimuli, and subtracted from the measured maximum following stimulation. For testing of the receptor antagonists, cells were pretreated for 20 min with the antagonists, followed by stimulation with the respective agonists at the previously determined EC 80 concentration. Values are expressed as percent signal of agonist.

### 4.19. Adenovirus Infection

Adenoviruses carrying GFP under different promotors (CMV, EF1, and UBIC) were obtained from Sirion Biotech (SB-S-AV-104-01, Matinsried, Germany). Macrophage progenitors were mixed with the virus directly before plating, or virus was added to the suspension cultures at an MOI of 10. GFP expression was assessed using automated microscopy (Operetta CLS, Perkin Elmer, Waltham, MA, USA) and high-content analysis software (Harmony, Perkin Elmer, Waltham, MA, USA).

### 4.20. Data Handling and Statistics

Unless otherwise mentioned, all data values are expressed as means ± standard deviation (SD). Unless otherwise indicated, experiments were performed at least three times (i.e., using three different cell preparations), with at least three technical replicates per condition. Statistical methods for analyzing the various data sets are indicated directly in the figure legends.

## 5. Patents

The method described here is also part of a patent application PCT/EP2020/064481.

## Supplementary Materials

Supplementary materials can be found at https://www.mdpi.com/1422-0067/21/13/4808/s1.

## Abbreviations

BMP4	Bone morphogenetic protein 4
cAMP	Cyclic adenosine monophosphate
EB	Embryoid body
FCS	Fetal calf serum
GFP	Green fluorescent protein
GFR	Growth factor reduced
GM-CSF	Granulocyte-macrophage colony-stimulating factor
GO	Gene ontology
IL-3	Interleukin 3
IFNγ	Interferon gamma
iPSC	Induced pluripotent stem cells
LPS	Lipopolysaccharide
M-CSF	Macrophage colony-stimulating factor
PBMC	Peripheral blood mononuclear cell
SCF	Stem cell factor
VEGF	Vascular endothelial growth factor

## References

[1] Wynn T.A., Chawla A., Pollard J.W. (2013). Macrophage biology in development, homeostasis and disease. Nature.

[2] Sica A., Larghi P., Mancino A., Rubino L., Porta C., Totaro M.G., Rimoldi M., Biswas S.K., Allavena P., Mantovani A. (2008). Macrophage polarization in tumour progression. Semin. Cancer Biol..

[3] Mantovani A., Biswas S.K., Galdiero M.R., Sica A., Locati M. (2012). Macrophage plasticity and polarization in tissue repair and remodelling. J. Pathol..

[4] Biswas S.K., Chittezhath M., Shalova I.N., Lim J.-Y. (2012). Macrophage polarization and plasticity in health and disease. Immunol. Res..

[5] Shapouri-Moghaddam A., Mohammadian S., Vazini H., Taghadosi M., Esmaeili S.-A., Mardani F., Seifi B., Mahommadi A., Afshari J.T., Sahebkar A. (2018). Macrophage plasticity, polarization, and function in health and disease. J. Cell Physiol..

[6] Tiwari R., Singh V., Barthwal M.K. (2008). Macrophages: An elusive yet emerging therapeutic target of atherosclerosis. Med. Res. Rev..

[7] Ginhoux F., Jung S. (2014). Monocytes and macrophages: Developmental pathways and tissue homeostasis. Nat. Rev. Immunol..

[8] Lee C.Z.W., Kozaki T., Ginhoux F. (2018). Studying tissue macrophages in vitro: Are iPSC-derived cells the answer?. Nat. Rev. Immunol..

[9] Haenseler W., Zambon F., Lee H., Vowles J., Rinaldi F., Duggal G., Houlden H., Gwinn K., Wray S., Luk K.C. (2017). Excess alpha-synuclein compromises phagocytosis in iPSC-derived macrophages. Sci. Rep..

[10] Douvaras P., Sun B., Wang M., Kruglikov I., Lallos G., Zimmer M., Terrenoire C., Zhang B., Gandy S., Schadt E. (2017). Directed Differentiation of Human Pluripotent Stem Cells to Microglia. Stem Cell Rep..

[11] Park E.K., Jung H.S., Yang H.I., Yoo M.C., Kim C., Kim K.S. (2007). Optimized THP-1 differentiation is required for the detection of responses to weak stimuli. Inflamm. Res..

[12] Schildberger A., Rossmanith E., Eichhorn T., Strassl K., Weber V. (2013). Monocytes, Peripheral Blood Mononuclear Cells, and THP-1 Cells Exhibit Different Cytokine Expression Patterns following Stimulation with Lipopolysaccharide. Mediat. Inflamm..

[13] Tsuchiya S., Yamabe M., Yamaguchi Y., Kobayashi Y., Konno T., Tada K. (1980). Establishment and characterization of a human acute monocytic leukemia cell line (THP-1). Int. J. Cancer.

[14] Takamatsu K., Ikeda T., Haruta M., Matsumura K., Ogi Y., Nakagata N., Uchino M., Ando Y., Nishimura Y., Senju S. (2014). Degradation of amyloid beta by human induced pluripotent stem cell-derived macrophages expressing Neprilysin-2. Stem Cell Res..

[15] Senju S., Haruta M., Matsumura K., Matsunaga Y., Fukushima S., Ikeda T., Takamatsu K., Irie A., Nishimura Y. (2011). Generation of dendritic cells and macrophages from human induced pluripotent stem cells aiming at cell therapy. Gene Ther..

[16] Karlsson K.R., Cowley S., Martinez F.O., Shaw M., Minger S.L., James W.S. (2008). Homogeneous monocytes and macrophages from human embryonic stem cells following coculture-free differentiation in M-CSF and IL-3. Exp. Hematol..

[17] Hong D., Ding J., Li O., He Q., Ke M., Zhu M., Liu L., Ou W.-B., He Y., Wu Y. (2018). Human-induced pluripotent stem cell-derived macrophages and their immunological function in response to tuberculosis infection. Stem Cell Res. Ther..

[18] Haenseler W., Sansom S.N., Buchrieser J., Newey S.E., Moore C.S., Nicholls F.J., Chintawar S., Schnell C., Antel J.P., Allen N.D. (2017). A Highly Efficient Human Pluripotent Stem Cell Microglia Model.Model Displays a Neuronal-Co-culture-Specific Expression Profile and Inflammatory Response. Stem Cell Rep..

[19] Ackermann M., Kempf H., Hetzel M., Hesse C., Hashtchin A.R., Brinkert K., Schott J.W., Haake K., Kühnel M.P., Glage S. (2018). Bioreactor-based mass production of human iPSC-derived macrophages enables immunotherapies against bacterial airway infections. Nat. Commun..

[20] Ginhoux F., Greter M., Leboeuf M., Nandi S., See P., Gokhan S., Mehler M.F., Conway S.J., Gascoigne N.R.J., Stanley E.R. (2010). Fate Mapping Analysis Reveals That Adult Microglia Derive from Primitive Macrophages. Science.

[21] Gomez-Perdiguero E., Klapproth K., Schulz C., Busch K., Azzoni E., Crozet L., Garner H., Trouillet C., De Bruijn M., Geissmann F. (2015). Tissue-resident macrophages originate from yolk sac-derived erythro-myeloid progenitors. Exp. Hematol..

[22] Hoeffel G., Ginhoux F. (2015). Ontogeny of Tissue-Resident Macrophages. Front. Immunol..

[23] Kierdorf K., Erny D., Goldmann T., Sander V., Schulz C., Gomez-Perdiguero E., Wieghofer P., Heinrich A., Riemke P., Hölscher C. (2013). Microglia emerge from erythromyeloid precursors via Pu.1- and Irf8-dependent pathways. Nat. Neurosci..

[24] Schulz C., Gomez-Perdiguero E., Chorro L., Szabo-Rogers H., Cagnard N., Kierdorf K., Prinz M., Wu B., Jacobsen S.E.W., Pollard J.W. (2012). A Lineage of Myeloid Cells Independent of Myb and Hematopoietic Stem Cells. Science.

[25] Sheng J., Ruedl C., Karjalainen K. (2015). Most Tissue-Resident Macrophages Except Microglia Are Derived from Fetal Hematopoietic Stem Cells. Immunity.

[26] Epelman S., LaVine K.J., Beaudin A.E., Sojka R.K., Carrero J.A., Calderon B., Brija T., Gautier E.L., Ivanov S., Satpathy A.T. (2014). Embryonic and adult-derived resident cardiac macrophages are maintained through distinct mechanisms at steady state and during inflammation. Immunity.

[27] Hashimoto D., Chow A., Noizat C., Teo P., Beasley M.B., Leboeuf M., Becker C.D., See P., Price J., Lucas D. (2013). Tissue-resident macrophages self-maintain locally throughout adult life with minimal contribution from circulating monocytes. Immunity.

[28] Buchrieser J., James W., Moore M.D. (2017). Human Induced Pluripotent Stem Cell-Derived Macrophages Share Ontogeny with MYB-Independent Tissue-Resident Macrophages. Stem Cell Rep..

[29] Ginhoux F., Guilliams M. (2016). Tissue-Resident Macrophage Ontogeny and Homeostasis. Immunity.

[30] Takata K., Kozaki T., Lee C.Z.W., Thion M.S., Otsuka M., Lim S., Utami K.H., Fidan K., Park D.S., Malleret B. (2017). Induced-Pluripotent-Stem-Cell-Derived Primitive Macrophages Provide a Platform for Modeling Tissue-Resident Macrophage Differentiation and Function. Immunity.

[31] Van Wilgenburg B., Browne C., Vowles J., Cowley S.A. (2013). Efficient, Long Term Production of Monocyte-Derived Macrophages from Human Pluripotent Stem Cells under Partly-Defined and Fully-Defined Conditions. PLoS ONE.

[32] Metsalu T., Vilo J. (2015). ClustVis: A web tool for visualizing clustering of multivariate data using Principal Component Analysis and heatmap. Nucleic Acids Res..

[33] Ernst O., Glucksam-Galnoy Y., Athamna M., Ben-Dror I., Ben-Arosh H., Levy-Rimler G., Fraser I.D.C., Zor T. (2019). The cAMP Pathway Amplifies Early MyD88-Dependent and Type I Interferon-Independent LPS-Induced Interleukin-10 Expression in Mouse Macrophages. Mediat. Inflamm..

[34] Gobejishvili L., Ghare S., Khan R., Cambon A., Barker D.F., Barve S., McClain C., Hill D. (2015). Misoprostol modulates cytokine expression through a cAMP pathway: Potential therapeutic implication for liver disease. Clin. Immunol..

[35] Jin Y., Liu Y., Nelin L.D. (2014). Extracellular Signal-regulated Kinase Mediates Expression of Arginase II but Not Inducible Nitric-oxide Synthase in Lipopolysaccharide-stimulated Macrophages. J. Biol. Chem..

[36] Leslie C.C. (2015). Cytosolic phospholipase A2: Physiological function and role in disease. J. Lipid Res..

[37] Negreiros-Lima G.L., Lima K.M., Moreira I.Z., Jardim B.L.O., Vago J., Galvão I., Teixeira L.C., Pinho V., Teixeira M.M., Sugimoto M.A. (2020). Cyclic AMP Regulates Key Features of Macrophages via PKA: Recruitment, Reprogramming and Efferocytosis. Cells.

[38] Qian X., Zhang J., Liu J. (2010). Tumor-secreted PGE2Inhibits CCL5 Production in Activated Macrophages through cAMP/PKA Signaling Pathway. J. Biol. Chem..

[39] Veremeyko T., Yung A.W.Y., Dukhinova M., Kuznetsova I.S., Pomytkin I., Lyundup A., Strekalova T., Barteneva N.S., Ponomarev E.D. (2018). Cyclic AMP Pathway Suppress Autoimmune Neuroinflammation by Inhibiting Functions of Encephalitogenic CD4 T Cells and Enhancing M2 Macrophage Polarization at the Site of Inflammation. Front. Immunol..

[40] Zhou M., Xu W., Wang J., Yan J., Shi Y., Zhang C., Ge W., Wu J., Du P., Chen Y. (2018). Boosting mTOR-dependent autophagy via upstream TLR4-MyD88-MAPK signalling and downstream NF-kappaB pathway quenches intestinal inflammation and oxidative stress injury. EBioMedicine.

[41] Chawla A. (2010). Control of macrophage activation and function by PPARs. Circ. Res..

[42] Coll R.C., Robertson A.A., Chae J.J., Higgins S.C., Muñoz-Planillo R., Inserra M.C., Vetter I., Dungan L.S., Monks B.G., Stutz A. (2015). A small-molecule inhibitor of the NLRP3 inflammasome for the treatment of inflammatory diseases. Nat. Med..

[43] Darling N.J., Toth R., Arthur J.S.C., Clark K. (2017). Inhibition of SIK2 and SIK3 during differentiation enhances the anti-inflammatory phenotype of macrophages. Biochem. J..

[44] Heming M., Gran S., Jauch S.L., Fischer-Riepe L., Russo A., Klotz L., Hermann S., Schäfers M., Roth J., Barczyk-Kahlert K. (2018). Peroxisome Proliferator-Activated Receptor-gamma Modulates the Response of Macrophages to Lipopolysaccharide and Glucocorticoids. Front. Immunol..

[45] Nuñez V., Alameda D., Rico D., Mota-Blanco R.A., Gonzalo P., Cedenilla M., Fischer T., Boscá L., Glass C.K., Arroyo A.G. (2010). Retinoid X receptor α controls innate inflammatory responses through the up-regulation of chemokine expression. Proc. Natl. Acad. Sci. USA.

[46] Wang S., Liu F., Tan K.S., Ser H., Tan L.T., Lee L., Tan W. (2020). Effect of (R)-salbutamol on the switch of phenotype and metabolic pattern in LPS-induced macrophage cells. J. Cell. Mol. Med..

[47] Brull M., Spreng A.-S., Gutbier S., Loser D., Krebs A., Reich M., Kraushaar U., Britschgi M., Patsch C., Leist M. (2020). Incorporation of stem cell-derived astrocytes into neuronal organoids to allow neuro-glial interactions in toxicological studies. ALTEX.

[48] Hasselmann J., Blurton-Jones M. (2020). Human iPSC-derived microglia: A growing toolset to study the brain’s innate immune cells. Glia.

[49] Cao X., van den Hil F.E., Mummery C.L., Orlova V.V. (2020). Generation and Functional Characterization of Monocytes and Macrophages Derived from Human Induced Pluripotent Stem Cells. Curr. Protoc. Stem Cell Biol..

[50] Zhang H., Xue C., Shah R., Bermingham K., Hinkle C.C., Li W., Rodrigues A., Tabita-Martinez J., Millar J.S., Cuchel M. (2015). Functional analysis and transcriptomic profiling of iPSC-derived macrophages and their application in modeling Mendelian disease. Circ. Res..

[51] Andreone B.J., Przybyla L., Llapashtica C., Rana A., Davis S.S., Van Lengerich B., Lin K., Shi J., Mei Y., Astarita G. (2020). Alzheimer’s-associated PLCγ2 is a signaling node required for both TREM2 function and the inflammatory response in human microglia. Nat. Neurosci..

[52] McQuade A., Coburn M.A., Tu C.H., Hasselmann J., Davtyan H., Blurton-Jones M. (2018). Development and validation of a simplified method to generate human microglia from pluripotent stem cells. Mol. Neurodegener..

[53] Fernandes H.J.R., Hartfield E.M., Christian H.C., Emmanoulidou E., Zheng Y., Booth H., Bogetofte H., Lang C., Ryan B., Sardi S.P. (2016). ER Stress and Autophagic Perturbations Lead to Elevated Extracellular α-Synuclein in GBA-N370S Parkinson’s iPSC-Derived Dopamine Neurons. Stem Cell Rep..

[54] Volpato V., Smith J., Sandor C., Ried J.S., Baud A., Handel A., Newey S.E., Wessely F., Attar M., Whiteley E. (2018). Reproducibility of Molecular Phenotypes after Long-Term Differentiation to Human iPSC-Derived Neurons: A Multi-Site Omics Study. Stem Cell Rep..

[55] Dobin A., Davis C.A., Schlesinger F., Drenkow J., Zaleski C., Jha S., Batut P., Chaisson M., Gingeras T.R. (2012). STAR: Ultrafast universal RNA-seq aligner. Bioinformatics.

[56] Andrews S. (2010). Fastqc: A Quality Control Tool for High Throughput Sequence Data.

[57] Ewels P., Magnusson M., Lundin S., Käller M. (2016). MultiQC: Summarize analysis results for multiple tools and samples in a single report. Bioinformatics.

[58] Li H., Handsaker B., Wysoker A., Fennell T., Ruan J., Homer N., Marth G., Abecasis G.R., Durbin R., 1000 Genome Project Data Processing Subgroup (2009). The Sequence Alignment/Map format and SAMtools. Bioinformatics.

[59] Mortazavi A., A Williams B., McCue K., Schaeffer L., Wold B. (2008). Mapping and quantifying mammalian transcriptomes by RNA-Seq. Nat. Methods.

