# Supplementary material for: Large-Scale Production of Human iPSC-Derived Macrophages for Drug Screening
Source: Int J Mol Sci. 2020 Jul 7;21(13):4808. doi: 10.3390/ijms21134808 (PMC7370446; doi:10.3390/ijms21134808)
Supplement: Supplementary file 1 [file ijms-21-04808-s001.zip › Supplementary Files/Supplementary Information Gutbier et al revised.pdf]

Large-scale production of human iPSC-derived macrophages for drug screening

S. Gutbier[1,3,4], F. Wanke[2], N. Dahm[1], A. Rümmelin[1,2], S. Zimmermann[1], K. Christensen[1], F. Köchl[3], A. Rautanen[3], K. Hatje[3], B. Geering[2], D. Zhang[3], M. Britschgi[4], S. A. Cowley[5], C. Patsch[1,6]

Supplementary Information

**Material**	**Supplier**	**Cat.No.**
**Plastic ware:**		
Aggrewell 800	STEMCELL Technologies	27865
B10, B15 , Cell disk	Falcon, VWR, Greiner	353003, 734-2322, 678101
Strainer 40 uM	Falcon	352360
**Coating:**		
Lam521	BioLamina	LN521-05
Growth factor reduced Matrigel	Corning	354230
**Media + Supplements**		
mTESR	STEMCELL Technologies	85850
XVIVO 15	Lonza	BE02-053Q
DMEM/F12	Gibco	12634-010
Glutamax	Gibco	35050-061
Penicillin/Streptomycin	Gibco	15140-122
Beta-Mercaptoethanol	Gibco	31350-010
PBS containing Ca/Mg (++)	Gibco	14040174
PBS w/o Ca/Mg	Gibco	14190250
Accutase	Innovative Cell Technologies	AT-104
**Growth factors/morphogens**		
Y27632 (Rock inhibitor)	Callbiochem	688000
hrVEGF(vascular endothelial growth factor),	R&D	293-VE
hrSCF (human stem cell factor)	R&D	235-SC
hrBMP4 (human recombinant bone morphogenic protein-4)	R&D	314-BP
hrIL-3 (interleukin 3)	Miltenyi Biotech	130-095-069
hrM-CSF (macrophage colony-stimulating factor)	Miltenyi Biotech	130-096-493
**FACS_QC Setup:**		
CD11b_APC, anti REA ab	Miltenyi Biotech	130-110-612
CD14-PE, antiREA	Miltenyi Biotech	130-110-577
CD68-APC Vio770, anti REA ab	Miltenyi Biotech	130-114-654
CD16_Vio PE770, anti REA ab	Miltenyi Biotech	130-113-956
Ki67-PEVio615, anti REA ab	Miltenyi Biotech	130-120-558
FOX-P3 buffer kit	Miltenyi Biotech	130-093-142
Auto MACS Running buffer or BSA/EDTA containing buffer	Miltenyi Biotech	130-091-221
AntiREA(I) ctrl -PEVio615	Miltenyi Biotech	130-107-771
Viobility 405/452	Miltenyi Biotech	130-109-816

FigS.1 Material list

day21	Lam521 IPS SFC840-03-01	Matrigel IPS SFC840-03-01
% pos CD14	73.5	-
% pos CD11b	65.3	-
% pos CD68	-	-
% pos Ki67	-	-
yield of cells	1.5x106	-
**day34**	**Lam521 IPS SFC840-03-01**	**Matrigel IPS SFC840-03-01**
% pos CD14	98.6	98.5
% pos CD11b	97.6	97.1
% pos CD68	96.9	95.7
% pos Ki67	3.65	2.4
yield of cells	36.5x106	1.2x106
**day41**	**Lam521 IPS SFC840-03-01**	**Matrigel IPS SFC840-03-01**
% pos CD14	97.3	98.5
% pos CD11b	98.2	98.7
% pos CD68	95.2	93.7
% pos Ki67	10.5	31
yield of cells	30x106	8.5x106

FigS.2 Comparison of myeloid factories derived from iPSC cultured either on LAM521 or Matrigel

Starting cultures of iPSC were either maintained on dishes coated with Lam521 or growth factor reduced matrigel. Embryoid body formation and EB replating was performed as described in materials and methods. Harvests from myeloid factories (B10 dishes) at different days of differentiation were compared in regards of yield and marker expression

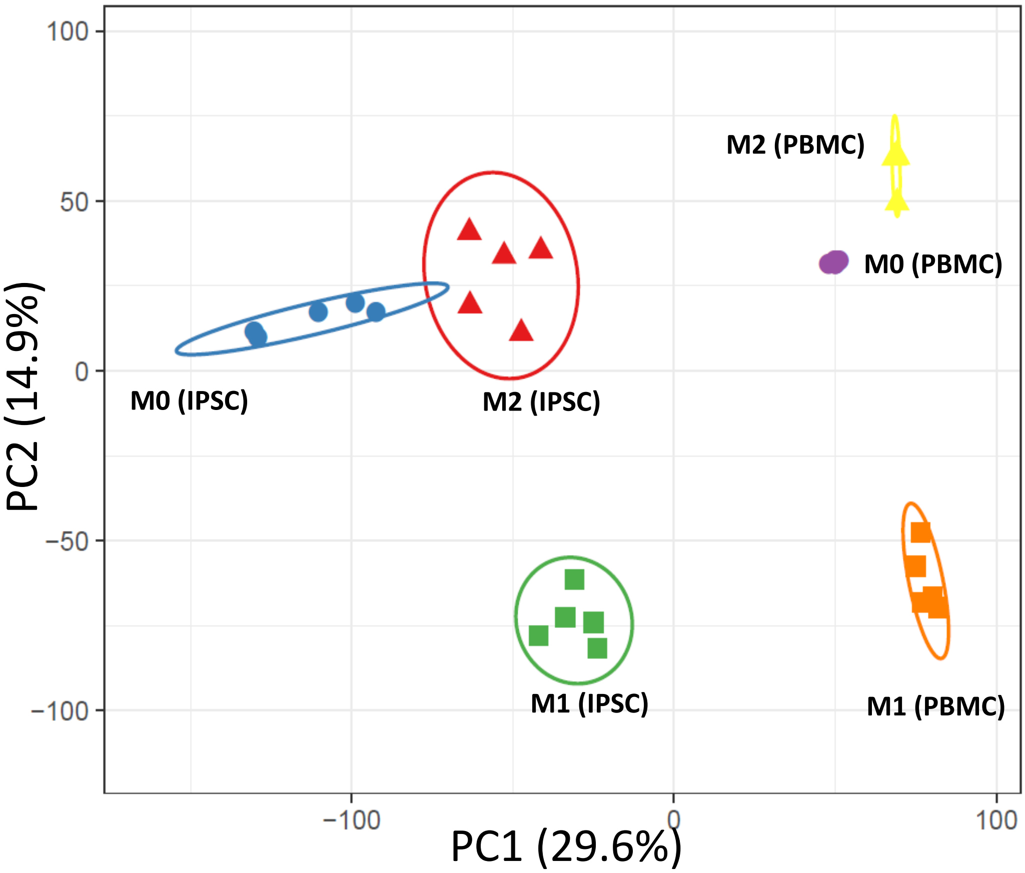

FigS.3 PCA of complete gene set covered by RNAseq.

Macrophages derived either from CD14 positive cells purchased from LONZA (single donor, 5 different vials thawed)or derived from iPSC (5 different differentiations from one iPSC line) via myeloid factories were polarized for 7 days. RNA was extracted and gene expression profile was assessed using RNAseq. A principal component analysis of the complete data set is shown.

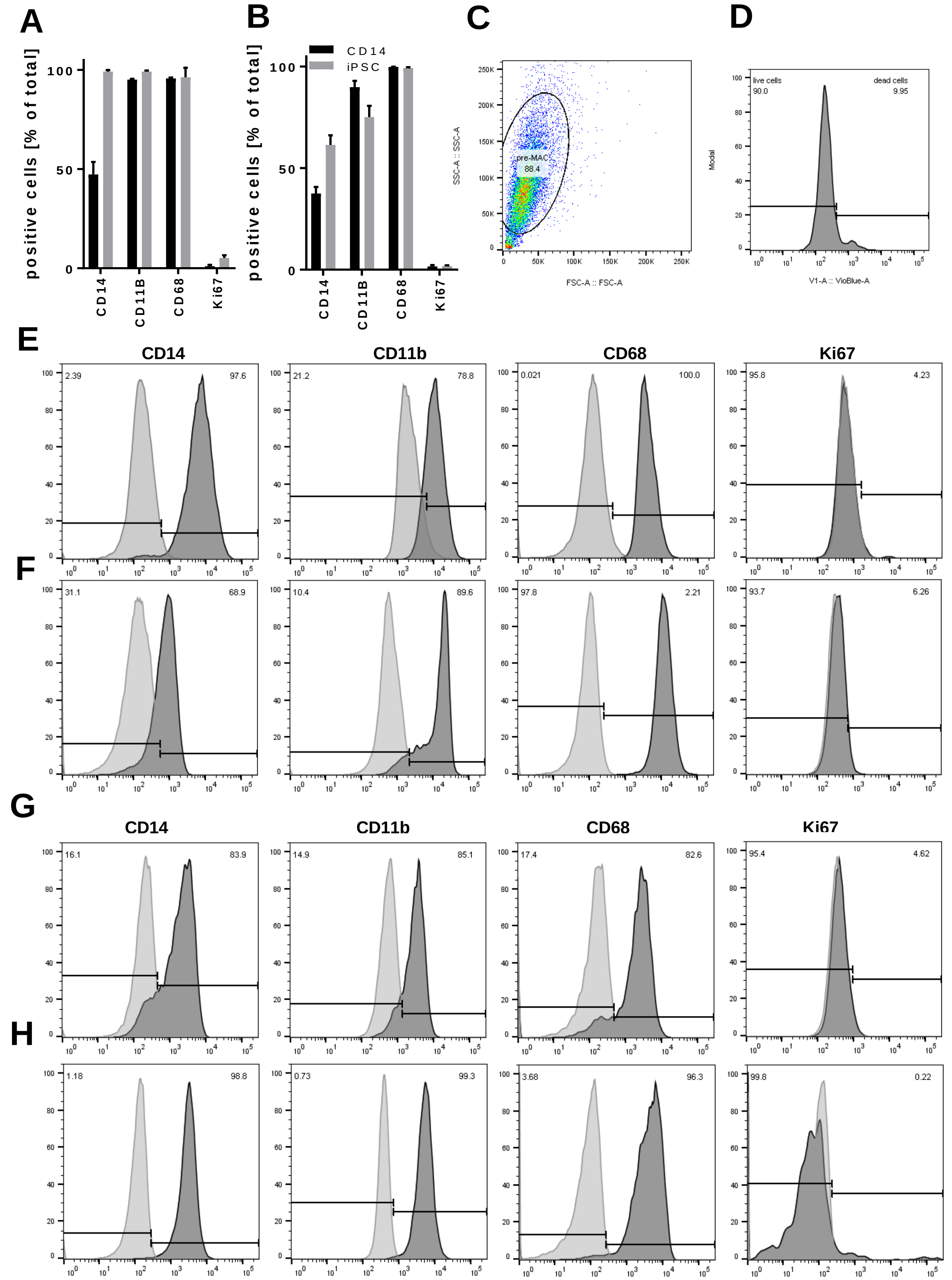

Fig. S4: Marker expression in PBMC derived monocytes and Macrophages

CD14 positive cells purchased from LONZA and macrophage progenitors and macrophages derived from iPSC were stained for CD14, CD68, CD11B and Ki67 an analyzed by flow cytometry. **A:** Marker expression in CD14 monocytes and macrophage progenitors derived from iPSC (n=3) **B:** Marker expression in CD14 derived macrophages (M0) and macrophage (M0) derived from iPSC (n=3) **C:** Gating strategy forward-sideward scatter **D:** Gating strategy viability for live stainings **E**: Representative FACS plots for macrophage progenitors derived from iPSC **F:** : Representative FACS plots for monocytes from LONZA **G**: Representative FACS plots for macrophages derived from iPSC **H:** : Representative FACS plots for macrophages derived from LONZA CD14 positive cells

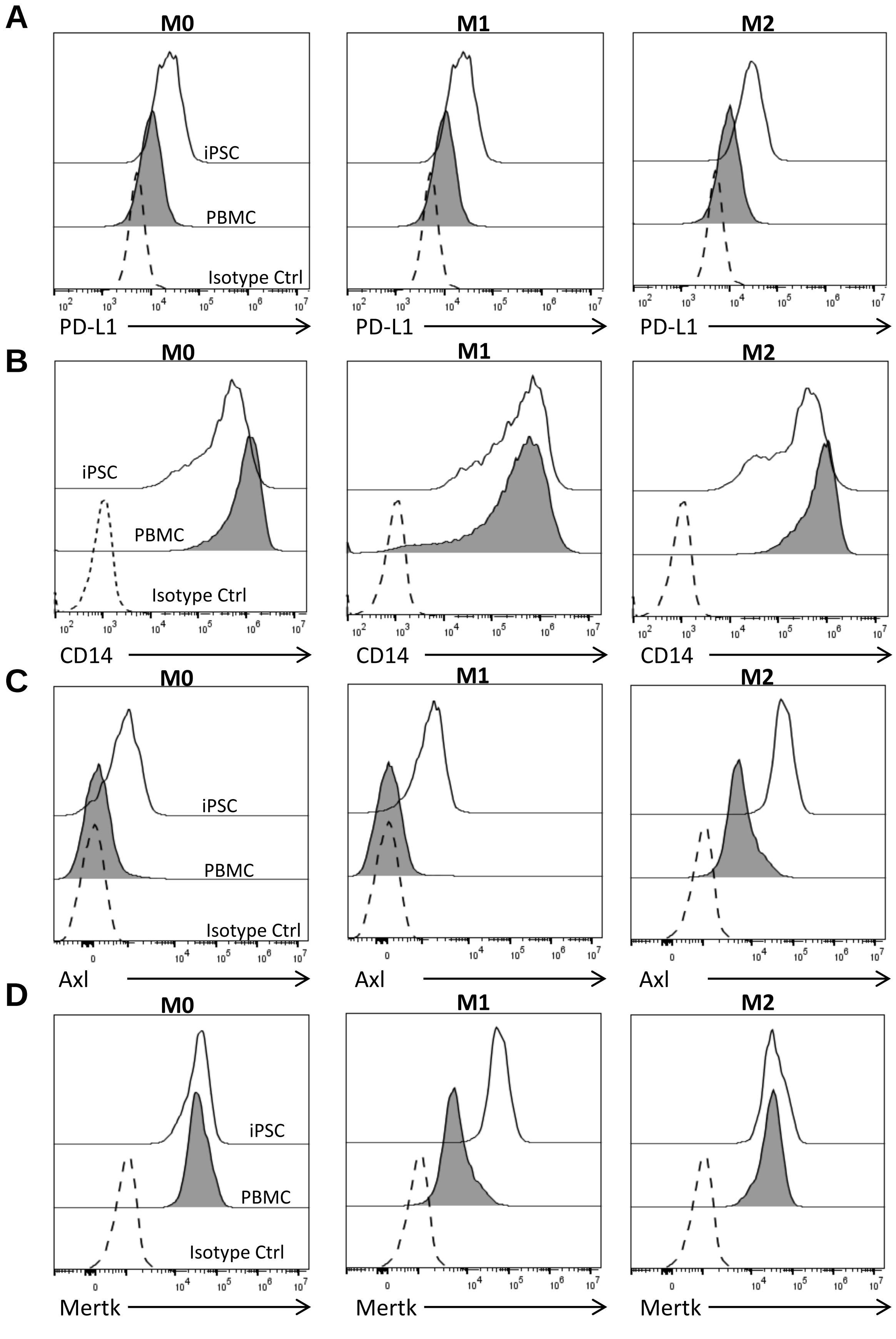

Fig. S5: Marker expression in IPSC and PBMC derived Macrophages

Macrophages derived from iPSC and PBMC were stained for PD-L1 CD14, AXL and Mertk and analyzed by flow cytometry. **A:** Intensity histogram of PD-L1 of macrophages derived from PBMC and iPSC **B:** Intensity histogram of CD14 macrophages derived from PBMC and iPSC **C:** Intensity histogram of AXL macrophages derived from PBMC and iPSC **D**: Intensity histogram of MERTK macrophages derived from PBMC and iPSC

Compound	MoA	Pathways	Rationale	Refrences
Forskolin	activator of adenylate cyclase	cAMP downstream pathways	investigation of GPCR downstream responses	PMID: 29422898;PMID 21097507
Dibuturyl cAMP	cAMP elevation	cAMP downstream pathways	investigation of GPCR downstream responses	PMID: 31935860
AZD8055	mTOR inhibitor	cAMP downstream pathways	investigation of GPCR downstream responses	PMID: 30170968
SB203580	p38 inhibitor	cAMP downstream pathways	investigation of GPCR downstream responses	PMID: 31148944
Darapladib	PLA2 inhibitor	cAMP downstream pathways	investigation of GPCR downstream responses	PMID: 25838312
H-89	PKA inhibitor	cAMP downstream pathways	investigation of GPCR downstream responses	PMID: 31935860
JSH-23	NFkB inhibitor	cAMP downstream pathways	investigation of GPCR downstream responses	PMID: 26408955
SCH772981	ERK1/2 inhibitor	cAMP downstream pathways	investigation of GPCR downstream responses	PMID: 25451938
Pioglitazone	PPARγ agonist	metabolic pathways	inflammatory modulation	PMID: 29867927; PMID: 20508200
9cis-Retinoicacid	RXR-agonist	ryanoid receptor signaling	inflammatory modulation	PMID: 20498053
Bexarotene	RXR-agonist	ryanoid receptor signaling	inflammatory modulation	PMID: 20498053
Salbutamol	β2 adrenergic receptor agonist	adrenergic signaling	inflammatory modulation	PMID: 31680470
HG-9-91-01	SIK inhibitor	salt inducible kinases	inflammatory modulation	PMID: 27920213
MCC950	NRLP3 inhibitor	inflammasome	inflammatory modulation	PMID: 25686105

Fig. S6: Rationale for compound selection

Compounds were selected by their targets being related to cAMP signaling or associated with direct immune modulatory properties.

Supplement zu Fig. 5: Cells can be used to identify modulators of phagocytosis

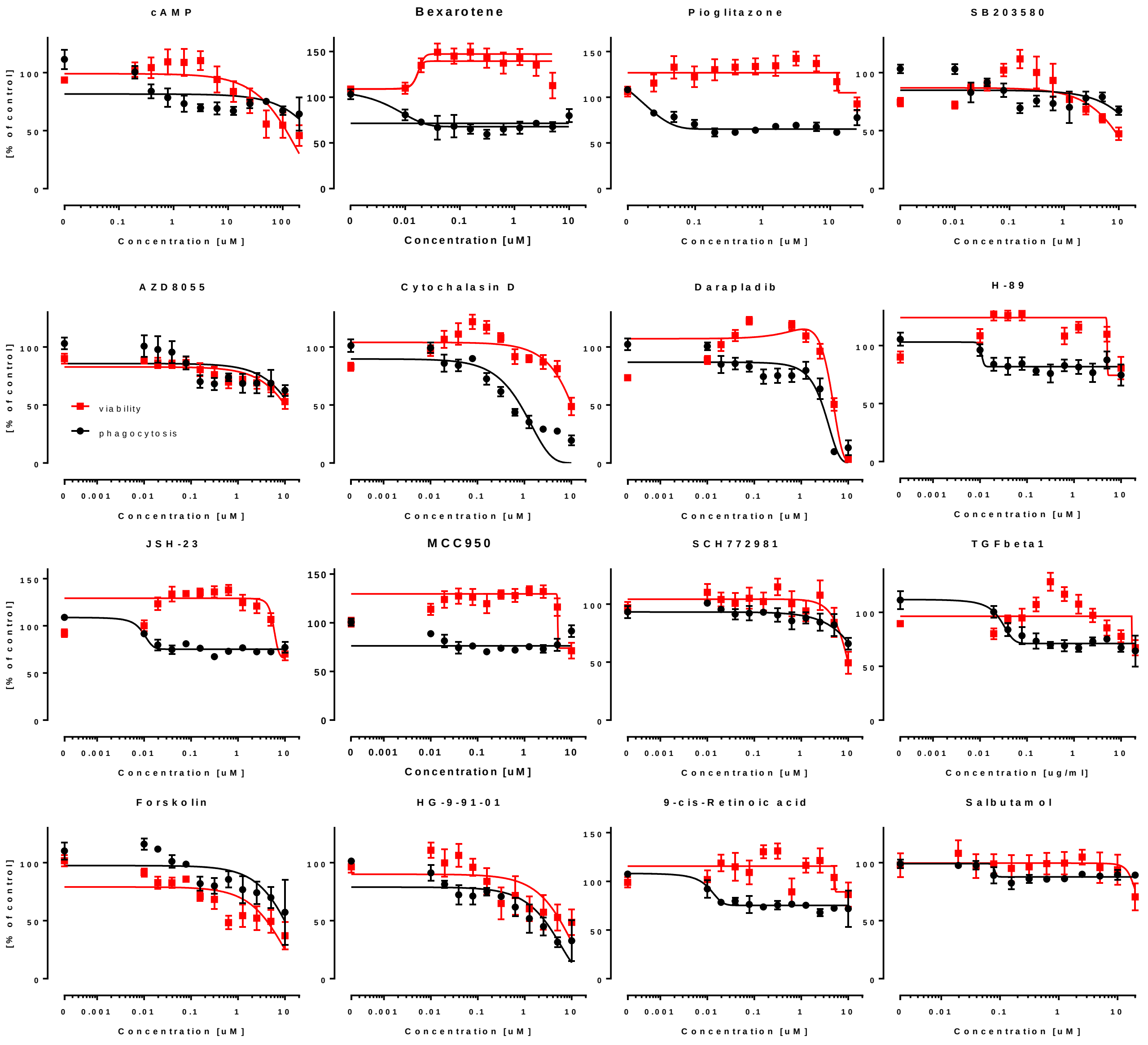

Fig. S7: Dose response curves for phagocytosis of zymosan

iPSC derived macrophage progenitors were seeded in 384 well plates at a density of 8000 cells per well and differentiated to M0 Macrophages for 6 days in XVIVO15 media containing M-CSF (100 ng/ml). Cells were pretreated with test compounds for 18 h and than exposed to Zymosan for 2h. Phagocytosis was assessed by high content imaging. Red dose response curves indicate total cell count per well normalized to solvent control. Black dose response curves indicate phagocytosis positive cells relative to solvent control. All values are indicated as mean +/- SEM of 3 independent experiments with 3 replicates per experiment.
