# Supplementary material for: Large-Scale Production of Human iPSC-Derived Macrophages for Drug Screening
Source: Int J Mol Sci. 2020 Jul 7;21(13):4808. doi: 10.3390/ijms21134808 (PMC7370446; doi:10.3390/ijms21134808)
Supplement: Supplementary file 1 [file ijms-21-04808-s001.zip › Supplementary Files/Supplementary videos Gutbier et al revised.pptx]

## Slide 1
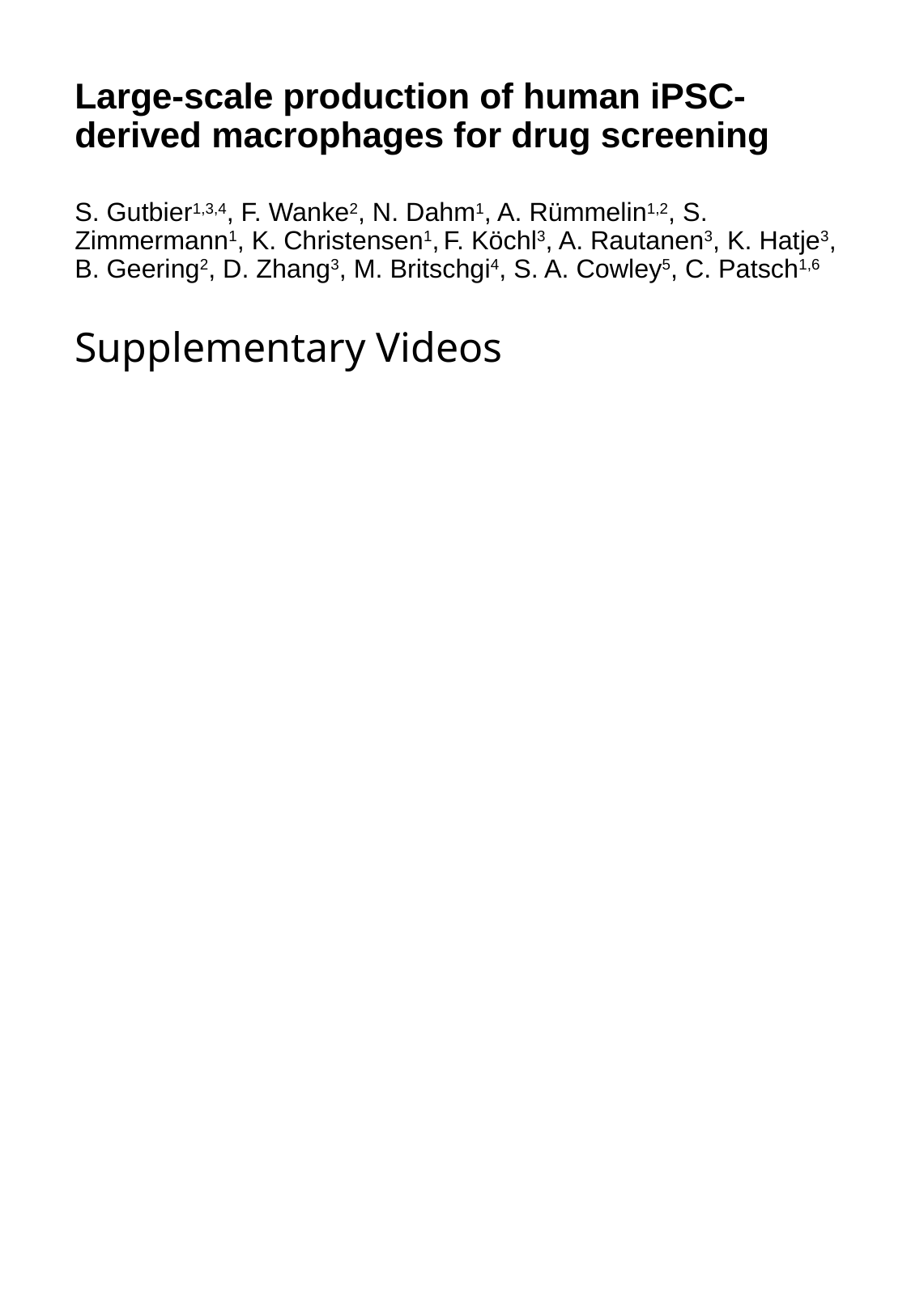

# Large-scale production of human iPSC-derived macrophages for drug screening S. Gutbier1,3,4, F. Wanke2, N. Dahm1, A. Rümmelin1,2, S. Zimmermann1, K. Christensen1, F. Köchl3, A. Rautanen3, K. Hatje3, B. Geering2, D. Zhang3, M. Britschgi4, S. A. Cowley5, C. Patsch1,6 Supplementary Videos

## Slide 2
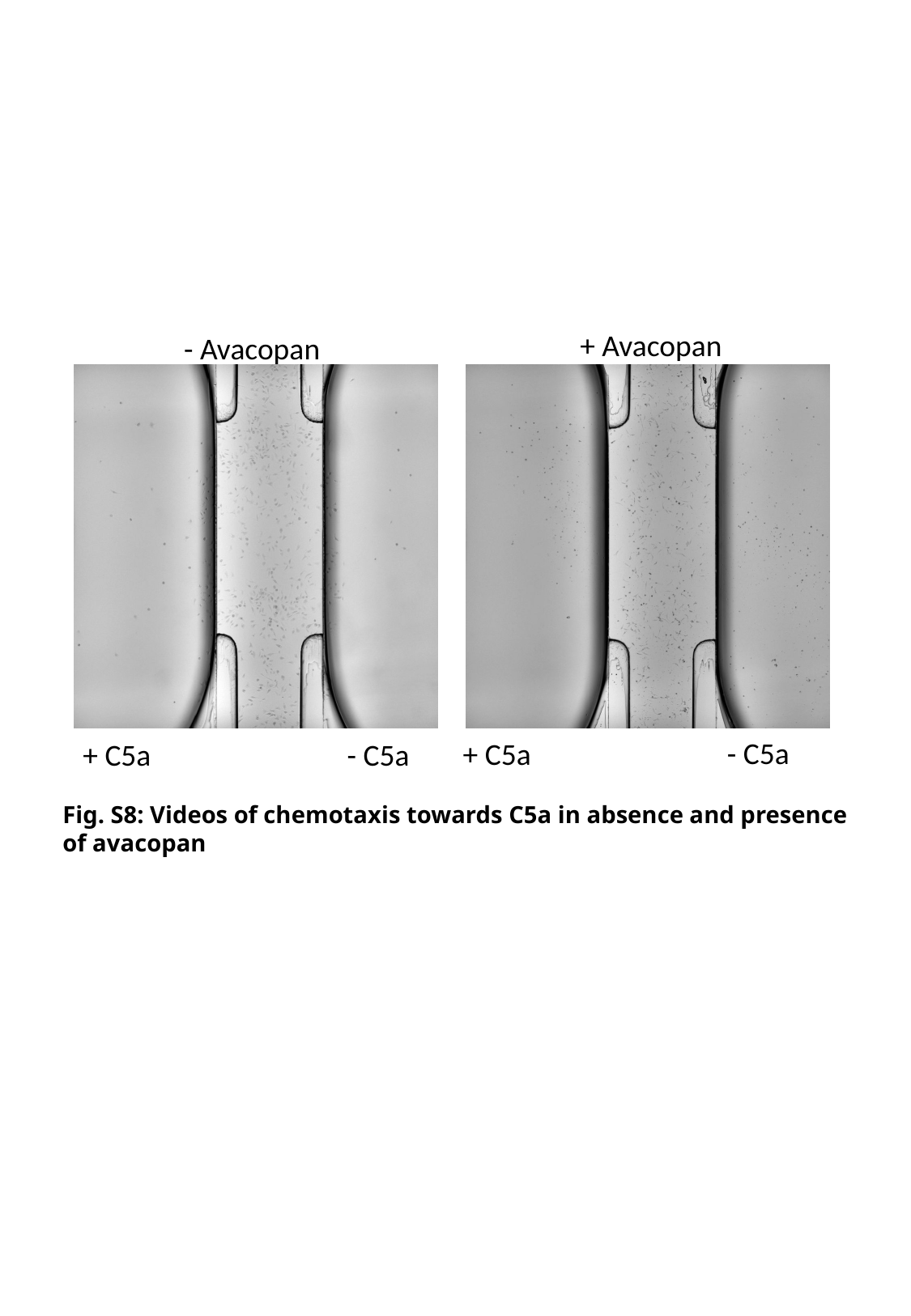

#
+ Avacopan
- Avacopan
Fig. S8: Videos of chemotaxis towards C5a in absence and presence of avacopan
- C5a
+ C5a
- C5a
+ C5a
